# Supplementary material for: Molecular Structures of Transcribing RNA Polymerase I
Source: Mol Cell. 2016 Dec 15;64(6):1135–43. doi: 10.1016/j.molcel.2016.11.013 (PMC5179497; doi:10.1016/j.molcel.2016.11.013)
Supplement: Document S1. Supplemental Experimental Procedures and Figures S1–S4 [file mmc1.pdf]

Molecular Cell, Volume *64*

Supplemental Information

Molecular Structures

of Transcribing RNA Polymerase I

Lucas Tafur, Yashar Sadian, Niklas A. Hoffmann, Arjen J. Jakobi, Rene Wetzel, Wim J.H. Hagen, Carsten Sachse, and Christoph W. Müller

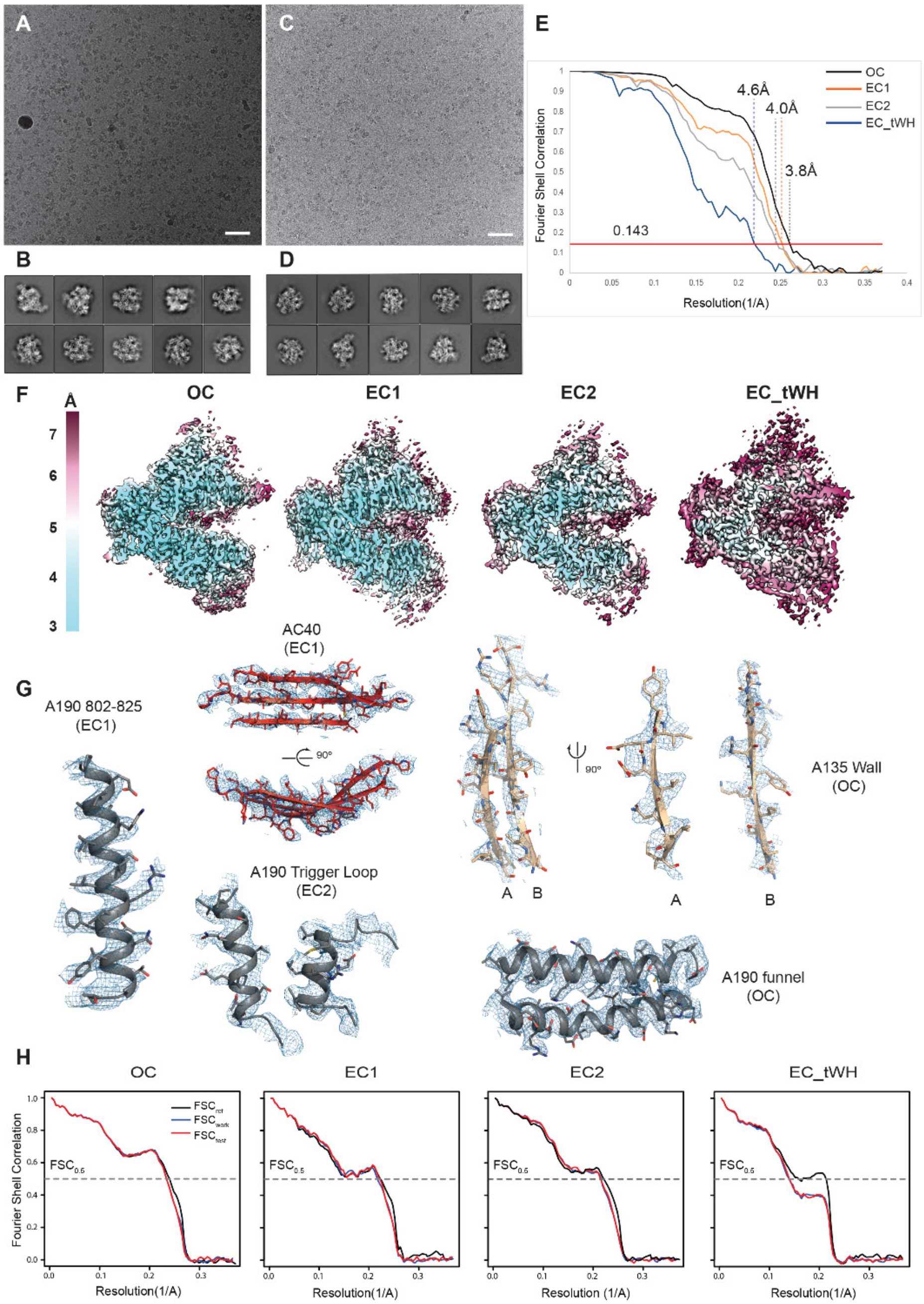

Figure S1. Related to Figure 1. Cryo-EM reconstructions of the Pol I OC, EC1, EC2 and EC_tWH. A, **C**, Representative micrographs for the Pol I OC, EC2, EC_tWH and for the EC1, respectively. Scale bar = 50 nm. **B**, **D**, Representative 2D class averages. **E**, FSC curves of the final reconstructions. The red line indicates the resolution cutoff according to the 0.143 criterion. **F,** The local resolution is displayed on a cross-section of the final electron microscopy maps. **G**, Representative electron microscopy densities of the EC, EC2 and OC. Features such as alpha-helical pitch, large side-chain density and beta-strand separation are discernible **H**, FSC curves calculated between the refined atomic model of OC, EC1, EC2 and EC_tWH, and the half map used in refinement (FSC_{work}) are shown in blue, those calculated between the refined atomic model and the second half map not used for refinement (FSC_{test}) in red. The FSC 0.5 is shown as dashed line. Close agreement between FSC_{work} and FSC_{test} and the absence of a sharp drop beyond the refinement target resolution indicate that no overfitting took place. As reference, the FSC between the refined atomic model and the map obtained from 3D reconstruction using the entire data set (FSC_{ref}) is also shown (black).

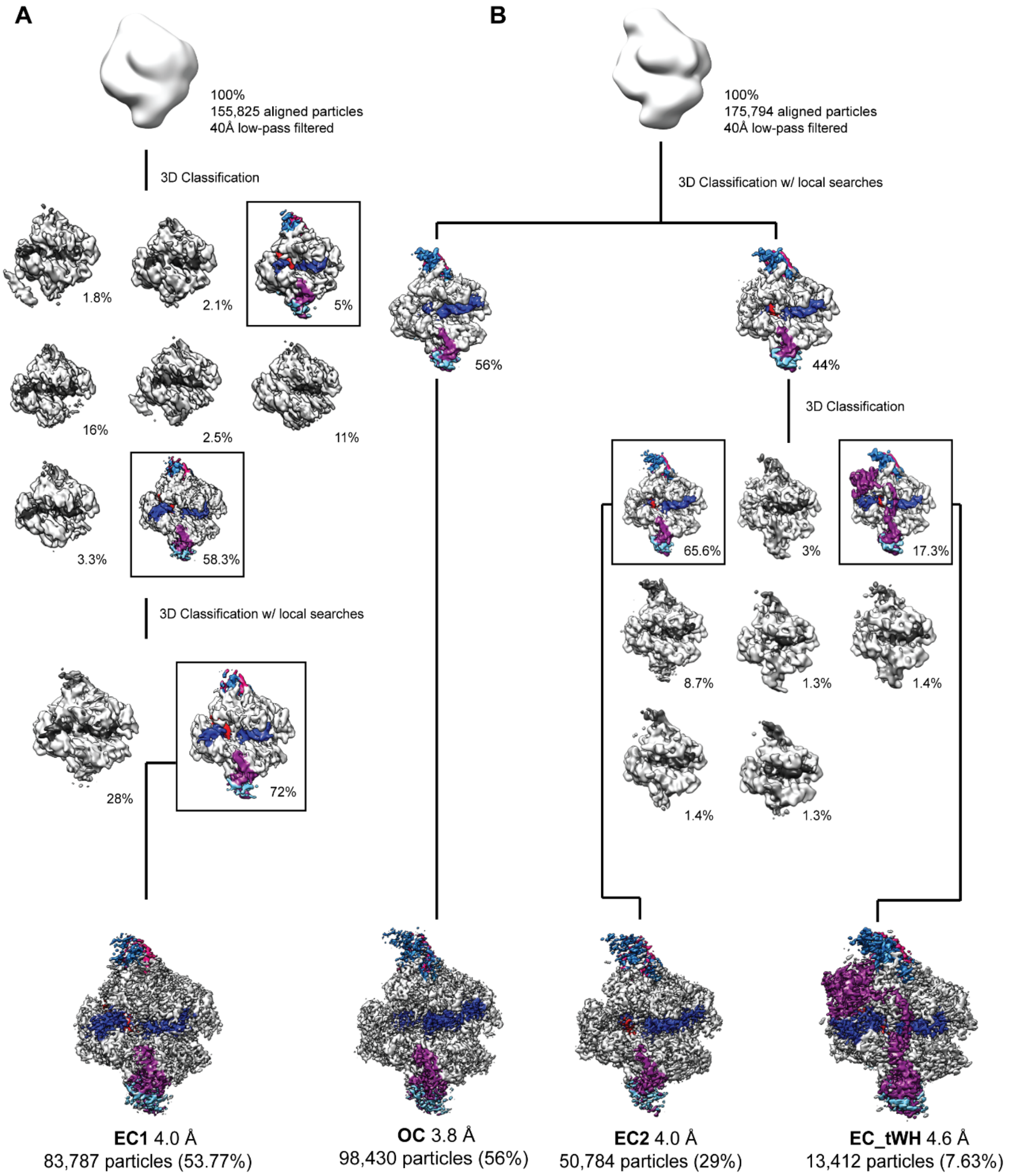

Figure S2. Related to Figure 1. Cryo-EM processing pipeline. A, Processing pipeline for the EC1 dataset. **B**, Processing pipeline for the OC, EC2 and EC_tWH dataset. Density has been colored according to the stalk subunits (A43, marine blue /A14, dark pink), heterodimer subunits (A49, purple /A34.5, lanthanum) and nucleic acids (DNA, blue; RNA, red). The percentage of particles included in each class are shown. Boxed, colored classes indicate classes which were selected for further processing. Discarded classes are shown in grey coloring. The final number of particles and the percentage from the initial aligned particles (100%, shown as the 40Å reference) in each reconstruction are shown.

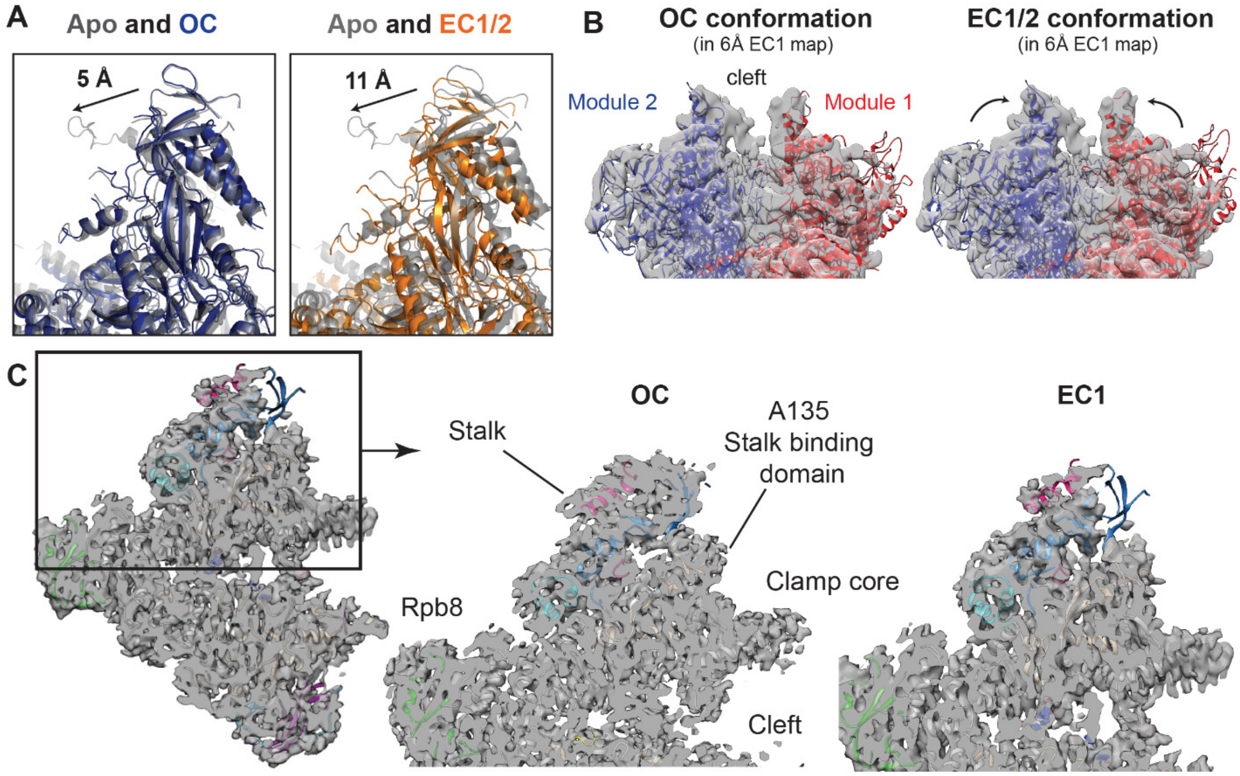

Figure S3. Related to Figure 1. Flexibility of the A43-A14 stalk. **A,** Movement of the stalk is coupled to closing of the cleft. The maximal distance moved relative to apo Pol I is indicated for the OC (left) and EC1/2 (right). **B**, Rigid-body fitting of the OC conformation into the EC1 map, filtered to 6 Å. Module 1 is shown in red and module 2 in blue. Each module fits into the density when moved independently (right) from the OC conformation (left). **C**, A slice of the EM density for the EC1 at a high threshold with the model is shown (left). At this threshold the anchored part of the stalk and the A135 stalk binding domain are seen in the density, but the distal part of the stalk is only visible in the OC. The close-up view of the boxed region is shown for the OC and the EC1 (right).

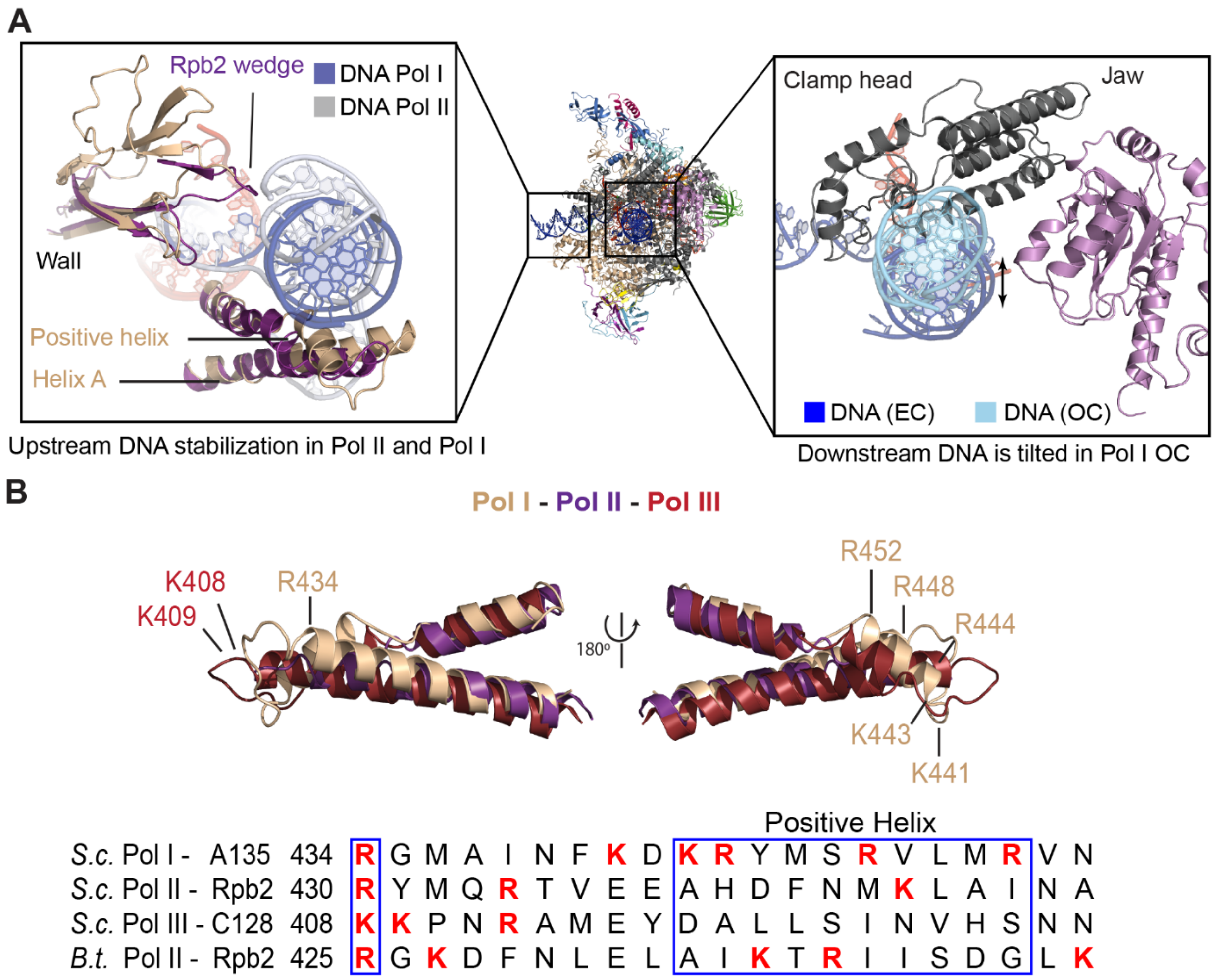

Figure S4. Related to Figure 2. Upstream and downstream DNA contacts in Pol I. A, **Left panel,** compared to Pol II (PDB: 5C4J), the upstream DNA is closer to the protrusion domain than to the wedge. Pol II is indicated in purple, and Pol I A135 subunit is in wheat. Pol I DNA is blue and Pol II DNA is in grey. The protrusion positive helix and helix A are indicated. **Right panel**, The downstream DNA in the OC is tilted towards the A190 clamp head domain and Rpb5 jaw interface, while the downstream DNA in the EC follows a straight path. **B,** Part of the protrusion domain of Pol I, II (PDB: 1Y1W) and III (PDB: 5FJ8) are superimposed. Positive residues in this region are highlighted and the corresponding structure-based sequence alignment is shown below. For comparison, the *Bos Taurus* Rpb2 sequence is aligned, showing that it has more positive residues than *Saccharomyces cerevisiae* Rpb2 and C128 in this region.

Supplemental Experimental Procedures

Protein purification

Pol I was purified from *Saccharomyces cerevisiae* strain SC1613 (also calledYPR110c, provided by Cellzome AG), with a C-terminal TAP-tag fused to AC40. Yeast cells were grown overnight in YPDA medium at 30 ºC and 180 r.p.m. under controlled conditions and collected at an OD_{600} of 5–6. All purification steps were performed at 4 ºC. The cell paste was re-suspended in a buffer containing 250 mM Tris-HCl, pH 7.5, 40% glycerol, 250 mM ammonium sulfate, 1 mM EDTA, 10 mM $MgCl_2$, 10 mM $ZnCl_2$,12 mM β-mercaptoethanol with protease inhibitors (Complete EDTA-free, Roche) and lysed with glass beads in a BeadBeater (BioSpec). The lysate was cleared by centrifugation at 14,000 r.p.m. for 1 h at 4 ºC and loaded on heparin-sepharose resin (GE Healthcare). The complex was eluted from the resin using high-salt buffer with 1 M ammonium sulfate and incubated with IgG Sepharose (GE-Healthcare) for 5 h. After washing the unbound proteins, IgG beads were incubated overnight at 4 ºC with tobacco etch virus (TEV) protease. TAP-tag cleaved Pol I was eluted and further purified by ionic exchange on a Mono-Q column (GE- Healthcare). Pure Pol I enzyme was concentrated and buffer exchanged to 7 mg/ml in 150 mM ammonium sulfate, 15 mM HEPES-NaOH pH 7.5 and 10 mM DTT. Aliquots were stored after flash-freezing in liquid nitrogen or used immediately for complex preparation.

Image processing

All processing steps were performed in RELION-1.4 (Scheres, 2012) unless noted otherwise. The contrast transfer function determination was performed using CTFFIND4 (Rohou and Grigorieff, 2015) and the Thon rings were visually inspected to select good micrographs. B-factor sharpening was performed through implementation in RELION-1.4 as described (Rosenthal and Henderson, 2003) prior to visualization. The resolution is reported according to the FSC 0.143 criterion after particle polishing in RELION. Local resolution of the maps was calculated using Blocres (Cardone et al., 2013).

<u>EC1</u>

Raw movie frames were gain corrected, Fourier cropped, and then aligned using UNBLUR (Grant and Grigorieff, 2015). Initially, approximately 10,000 particles were manually picked using semi-automated particle picking in EMAN2 and extracted with a 200 pixel box size (Tang et al., 2007). Particles were subjected to an initial 2D classification and good classes were selected as templates for the auto-picking procedure in RELION. Autopicked particles (342,189) were subjected to two rounds of 2D classification. Pooled particles (155,825) were aligned using an auto-refine run against the crystal structure of apo Pol I (PDB: 4C3I) low pass filtered to 60 Å and then subjected to 3D classification, using the aligned reconstruction as a reference. Two abundant classes that showed good alignment (98,842 particles, 63.3%) were selected and subjected to 3D classification with restrained angular searches to further sub-classify conformational differences between the retained particles, leading to a class (83,787 particles) which was refined to 4.0 Å. Further classification resulted in maps with the same conformation but lower resolvability. We observe high flexibility for previously known mobile modules. In particular, the A190 Jaw domain, the dimerization interface of the A49-A34.5 heterodimer and the N-terminal region of A12.2 are highly flexible but present in all the reconstruction at lower than the nominal resolution. However, the OB domain of the stalk subunit is completely flexible in the EC1 and could not be resolved by focusing the classification. We attribute this flexibility to movement of the modules during transcription elongation.

<u>OC, EC2, EC_tWH</u>

The movie frames were processed on-the-fly during data acquisition with SerialEM to motion-correct and sum the frames (Li et al., 2015). Approximately 30,000 particles were picked semi-automatically in EMAN2 and extracted with a 176 pixel box size (Tang et al., 2007). Particles were 2D-classified and good classes were used as templates for autopicking. 867,673 autopicked particles were extracted and sorted with 2D classification. Only a minor fraction showed density for CF and Rrn3, while the majority of particles represented Pol I bound to the transcription scaffold. All the particles selected after 2D classification (508,049 particles) were first refined using an auto-refine run against the Apo Pol I crystal structure (PDB: 4C3I) low pass filtered to 40 Å. Subsequently, aligned particles were subjected to 3D classification resulting in one major class (175,794 particles). A next round of 3D classification with restrained angular searches produced two classes: one complex with density for the RNA and a weak extra density (class 1, 44%), and one class without apparent RNA density and with the C-terminal region of

A12.2 (class 2, 56%). The latter was refined to 3.8 Å (OC, 98,430 particles). Further classification of class 2 did not improve the transcription bubble density or resolvability, and no different conformations were observed. Class 1 was further sub-classified and two major classes were refined to 4.0 Å (EC2, 50,784 particles) and 4.6 Å (EC_tWH, 13,412 particles). Additional classification of these classes reduced map quality, probably because of a reduced particle number.

Model building and refinement

For model building of the EC1, EC2 and the OC, the apo Pol I structure (PDB: 4C3I) was used as a starting model. Maps were filtered to different resolution ranges and sharpened with varying B-factors for better interpretation of the density. Subdomains were rigid body fitted in UCSF Chimera (Pettersen et al., 2004) to a 6 Å low pass filtered map. Fitted models were inspected and corrected manually in COOT (Emsley and Cowtan, 2004). For flexible areas in the EC1 (A49-A34.5, A12.2 N-ter, A190 Jaw, distal end of A43-A14), the backbone was rigid body fitted based on the OC or EC2 map which showed better density in these areas and not further built. For the EC_tWH, the crystal structure of the A49 tWH (PDB: 3NFI) and the EC2 model were rigid body fitted in UCSF Chimera to a 8 Å low-pass filtered density. Only three extra residues were built into the tWH crystal structure (residues 182-184). An initial DNA model for the EC1 was built based on a similar transcription bubble (PDB: 5FLM) and modified accordingly. A similar approach was used for building the DNA in the OC, which was used for the EC2 and EC_tWH. Models were real-space refined against the respective maps using a scripted workflow based on CCP4 and PHENIX/cctbx (Adams et al., 2010) libraries essentially as described previously (Fromm et al., 2015; Hoffmann et al., 2015; Hoffmann et al., 2016). Secondary structure restraints were updated at every refinement cycle using conformation analysis based on virtual dihedrals (Williams et al., 2013). We applied additional resolution-dependent restraints for poor map regions using local resolution estimates as described before (Hoffmann et al., 2015). Zinc binding site geometries were restrained to reference distance and angle distributions according to Harding (Harding, 2001; Harding, 2006). Each round of model optimization was evaluated by computing the real-space cross-correlation (RSCC) between experimental map and a map calculated from the model coordinates using B-factor-weighted structure factors from the model coordinates applying electron atomic form factors (Colliex et al, 2006). Individual isotropic atomic displacement parameters (ADPs) were refined by optimizing the real-space correlation between model and experimental map and fitting to the computed RSCC profile. The EC1, EC2, EC_tWH and OC models were refined against the full map reconstructions. We perturbed the models by random atom displacements of 0.4 Å followed by re-refinement against one of the respective half maps (work map). Overfitting of the models was then assessed by calculating the Fourier shell correlation against the work map (FSCwork) and the independent test map (FSCtest). For the OC model, the catalytic loop of A12.2 (residues 99-108) was included in the refinement and deleted afterwards.

Supplemental References

Adams, P.D., Afonine, P.V., Bunkoczi, G., Chen, V.B., Davis, I.W., Echols, N., Headd, J.J., Hung, L.W., Kapral, G.J., Grosse-Kunstleve, R.W.*, et al.* (2010). PHENIX: a comprehensive Python-based system for macromolecular structure solution. Acta Crystallogr D Biol Crystallogr *66*, 213-221.

Cardone, G., Heymann, J.B., and Steven, A.C. (2013). One number does not fit all: mapping local variations in resolution in cryo-EM reconstructions. J Struct Biol *184*, 226-236.

Colliex, C., Cowley, J.M., Dudarev, S.L., Fink, M., Gjønnes, J., Hilderbrandt, R., Howie, A., Lynch, D.F., Peng, L.M., Ren, G., et al. (2006). Electron diffraction. In International Tables for Crystallography (John Wiley & Sons, Ltd).

Fromm, S.A., Bharat, T.A., Jakobi, A.J., Hagen, W.J., and Sachse, C. (2015). Seeing tobacco mosaic virus through direct electron detectors. J Struct Biol *189*, 87-97.

Grant, T., and Grigorieff, N. (2015). Measuring the optimal exposure for single particle cryo-EM using a 2.6 A reconstruction of rotavirus VP6. Elife *4*, e06980.

Harding, M.M. (2001). Geometry of metal-ligand interactions in proteins. Acta Crystallogr D Biol Crystallogr *57*, 401-411.

Harding, M.M. (2006). Small revisions to predicted distances around metal sites in proteins. Acta Crystallogr D Biol Crystallogr *62*, 678-682.

Hoffmann, N.A., Jakobi, A.J., Vorlander, M.K., Sachse, C., and Muller, C.W. (2016). Transcribing RNA polymerase III observed by electron cryomicroscopy. FEBS J *283*, 2811-2819.

Li, X., Zheng, S., Agard, D.A., and Cheng, Y. (2015). Asynchronous data acquisition and on-the-fly analysis of dose fractionated cryoEM images by UCSFImage. J Struct Biol *192*, 174-178.

Rohou, A., and Grigorieff, N. (2015). CTFFIND4: Fast and accurate defocus estimation from electron micrographs. J Struct Biol *192*, 216-221.

Rosenthal, P.B., and Henderson, R. (2003). Optimal determination of particle orientation, absolute hand, and contrast loss in single-particle electron cryomicroscopy. J Mol Biol *333*, 721-745.

Tang, G., Peng, L., Baldwin, P.R., Mann, D.S., Jiang, W., Rees, I., and Ludtke, S.J. (2007). EMAN2: an extensible image processing suite for electron microscopy. J Struct Biol *157*, 38-46.
