# Supplementary material for: Molecular Structures of Transcribing RNA Polymerase I
Source: Mol Cell. 2016 Dec 15;64(6):1135–43. doi: 10.1016/j.molcel.2016.11.013 (PMC5179497; doi:10.1016/j.molcel.2016.11.013)
Supplement: Document S2. Article plus Supplemental Information [file mmc2.pdf]

Short Article

Molecular Cell

Molecular Structures of Transcribing RNA Polymerase I

Graphical Abstract

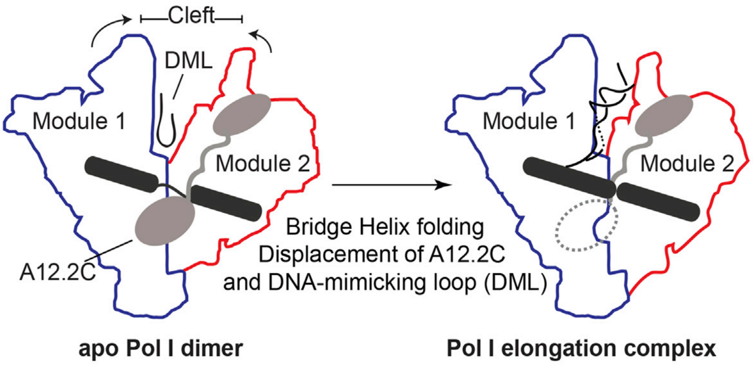

Authors

Lucas Tafur, Yashar Sadian, Niklas A. Hoffmann, ..., Wim J.H. Hagen, Carsten Sachse, Christoph W. Müller

Correspondence

cmueller@embl.de

In Brief

Tafur et al. present cryo-EM structures of transcribing Pol I that reveal the conformational changes required for rendering the enzyme active. During elongation, the DNA-binding cleft narrows, the bridge helix folds, and the A12.2 C-terminal domain is excluded from the active site.

Highlights

- Pol I gradually closes its DNA-binding cleft during elongation complex formation
- Pol I bridge helix folds into a conformation similar to that in other RNA polymerases
- During elongation, the Pol I A12.2 C-terminal domain is excluded from the cleft
- A49 tandem winged helix domain contacts upstream DNA, similar to TFIIE

Accession Numbers

5M5W
5M5X
5M5Y
5M64

Tafur et al., 2016, Molecular Cell *64*, 1135–1143
December 15, 2016 © 2016 The Author(s). Published by Elsevier Inc.
http://dx.doi.org/10.1016/j.molcel.2016.11.013

CellPress

Molecular Structures of Transcribing RNA Polymerase I

Lucas Tafur,[1] Yashar Sadian,[1] Niklas A. Hoffmann,[1] Arjen J. Jakobi,[1,2] Rene Wetzel,[1] Wim J.H. Hagen,[1] Carsten Sachse,[1] and Christoph W. Müller[1,3,*]
[1]European Molecular Biology Laboratory (EMBL), Structural and Computational Biology Unit, Meyerhofstrasse 1, 69117 Heidelberg, Germany
[2]European Molecular Biology Laboratory (EMBL), Hamburg Unit, Notkestrasse 85, 22607 Hamburg, Germany
[3]Lead Contact
*Correspondence: cmueller@embl.de
http://dx.doi.org/10.1016/j.molcel.2016.11.013

SUMMARY

RNA polymerase I (Pol I) is a 14-subunit enzyme that solely synthesizes pre-ribosomal RNA. Recently, the crystal structure of apo Pol I gave unprecedented insight into its molecular architecture. Here, we present three cryo-EM structures of elongating Pol I, two at 4.0 Å and one at 4.6 Å resolution, and a Pol I open complex at 3.8 Å resolution. Two modules in Pol I mediate the narrowing of the DNA-binding cleft by closing the clamp domain. The DNA is bound by the clamp head and by the protrusion domain, allowing visualization of the upstream and downstream DNA duplexes in one of the elongation complexes. During formation of the Pol I elongation complex, the bridge helix progressively folds, while the A12.2 C-terminal domain is displaced from the active site. Our results reveal the conformational changes associated with elongation complex formation and provide additional insight into the Pol I transcription cycle.

INTRODUCTION

The eukaryotic genome is transcribed by three RNA polymerases. RNA polymerase I (Pol I) transcribes a single pre-RNA gene, which is later processed in yeast into 5.8S, 18S, and 25S rRNAs. Pol II transcribes all mRNAs, and Pol III transcribes small structured RNAs such as tRNAs, 5S rRNA, and U6 snRNA. High transcriptional activity of Pol I (up to 60% of total transcription) is required for ribosome synthesis (Warner, 1999), and misregulation of Pol I transcription has been linked to different types of cancer (Drygin et al., 2010; Grummt, 2003; Poortinga et al., 2015). Accordingly, several small-molecule compounds that target Pol I and its transcription machinery are currently being tested as potential anti-cancer drugs (Colis et al., 2014; Drygin et al., 2011). Pol I is a 14-subunit enzyme that shares five subunits with Pol II and III (Rpb5, Rpb6, Rpb8, Rpb10, and Rpb12) that together with subunits A190, A135, AC40, AC19, and A12.2 form the core. Four additional subunits, the heterodimeric A43-A14 stalk, and the A49-A34.5 heterodimer complete the enzyme. While the overall organization of subunits is similar in all eukaryotic Pols, Pol I and III have incorporated Pol II general transcription factor-like subunits during evolution (Vannini and Cramer, 2012). The crystal structure of apo Pol I (Engel et al., 2013; Fernández-Tornero et al., 2013), as well as the cryoelectron microscopy (cryo-EM) structures of apo and transcribing Pol III (Hoffmann et al., 2015), has provided further insight into the mechanisms and evolution of eukaryotic RNA polymerases. In addition, cryo-EM structures of Pol I bound to the Pol I-specific factor Rrn3 have been reported (Engel et al., 2016; Pilsl et al., 2016). Apo Pol I was crystallized as a dimer in two independently solved structures, with each monomer showing a wide DNA-binding cleft (Engel et al., 2013; Fernández-Tornero et al., 2013). Dimerization of Pol I was mediated by the C-terminal region of stalk subunit A43, which inserted into the cleft of the neighboring Pol I. Additionally, the Pol I bridge helix (BH), a central, conserved element of the active center, was seen to be unwound in its middle region. Straight and bent BH conformations have been observed in bacterial Pol (Tuske et al., 2005), and limited movements of the BH during translocation have also been observed in Pol II (Brueckner and Cramer, 2008; Wang et al., 2006), consistent with complementary mutagenesis and molecular dynamics studies in different RNA polymerases (reviewed in Weinzierl, 2011). Interestingly, an acidic loop was observed in the DNA-binding cleft of Pol I, occupying a position similar to the DNA (“DNA-mimicking loop” or “expander”), while the C-terminal tandem winged helix domain (tWH) of A49 was absent in the complex. Overall, it was apparent that the apo Pol I crystal form, a dimer with a wide and occluded cleft, was incompatible with transcription elongation. However, dimerization has also been repeatedly observed in solution, suggesting that it might play a regulatory role (Bischler et al., 2002; Milkereit et al., 1997; Pilsl et al., 2016).

Here, we present cryo-EM structures of a Pol I open complex (OC) at 3.8 Å resolution; two fully engaged Pol I elongation complexes (EC1/2) bound to two different transcription scaffolds, both at 4.0 Å resolution; and one EC showing the A49 tWH at 4.6 Å resolution (EC_tWH). The different cryo-EM structures reveal closing of the DNA-binding cleft, folding of the BH, movement of the protrusion, and displacement of the A12.2 C-terminal domain from the active site. Comparison with the apo Pol I crystal structure and between the different cryo-EM structures reveals conformational rearrangements that mediate the gradual commitment of Pol I from transcription initiation to elongation and provide a framework by which this transition is promoted.

This is an open access article under the CC BY license (http://creativecommons.org/licenses/by/4.0/).

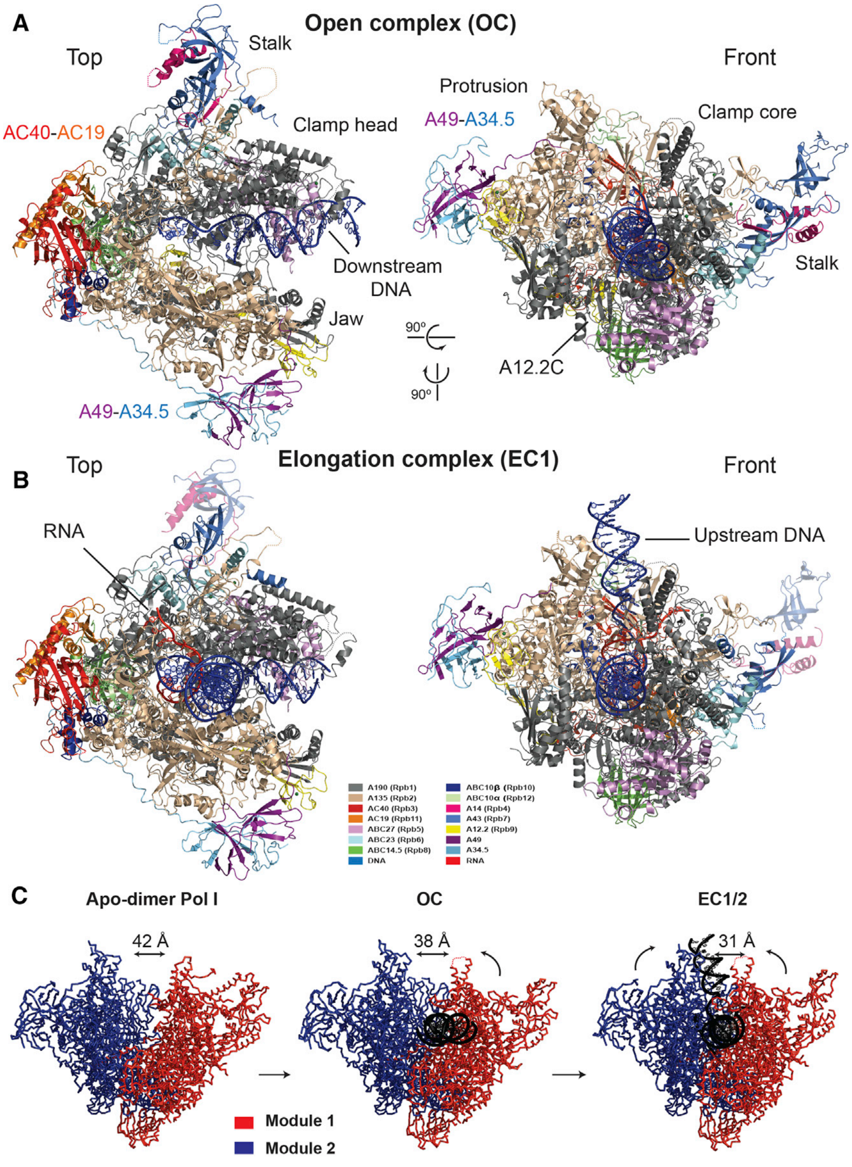

Figure 1. Cryo-EM Structures of Open Complex and Elongating RNA Polymerase I

(A) Top (left) and front (right) views of the open complex (OC).

(B) Top (left) and front (right) views of the elongation complex (EC1). Subunits are colored according to the labels on the box, and the corresponding Pol II subunits are shown in parentheses. Zn^{2+} ions are represented by green spheres. The distal part of the stalk in the EC1 is flexible and is shown for illustration purposes with weaker coloring.

(C) Schematic representation of modules 1 (red) and 2 (blue) after DNA binding. The distances above the cleft of apo Pol I (dimer), OC, and EC1/2 indicate the distance between the Cα atoms of subunit A135 residue 423 and A190 residue 429 located in the protrusion and clamp core domains, respectively.

See also Figures S1–S3 and Table 1.

RESULTS AND DISCUSSION

DNA-Binding Cleft of Pol I Closes during Elongation Complex Formation

To investigate the structural changes that Pol I undergoes upon initiation and elongation, we assembled two Pol I complexes with different transcription scaffolds: a 38-bp transcription scaffold containing an 11-nt transcription bubble and a 20-nt RNA oligonucleotide, as previously described (Hoffmann et al., 2015), and a longer 70-bp transcription scaffold containing the wild-type rDNA promoter sequence with a 15-nt transcription bubble and a 10-nt RNA oligonucleotide (Experimental Procedures). Cryo-EM data were collected on an FEI Titan Krios with a Gatan K2 Summit direct electron detector. For the 38-bp transcription scaffold, only one reconstruction of elongating Pol I complex at 4.0 Å resolution (EC1) was obtained (Figures 1, S1, and S2, available online). In contrast, three different complexes were sub-classified from the sample with the 70-bp transcription scaffold: a binary complex in which Pol I is bound to DNA but that does not show density for RNA that we therefore denote an open complex (OC) at 3.8 Å resolution (Figure 1), an elongation complex (EC2) at 4.0 Å resolution, and a minor fraction of particles in an EC conformation that showed additional density corresponding to the A49 tWH at 4.6 Å resolution (EC_tWH) (Figures S1 and S2).

In all reconstructions, the Pol I core, including its active center, is well defined, allowing unambiguous tracing of the main chain and depicting clear densities for most of the side chains (except for EC_tWH), while peripheral subunits like the A43-A14 stalk and the A49-A34.5 heterodimer and flexible loops show weaker densities (Figures S1F and S1G). We rigid-body fitted domains of the apo Pol I crystal structure (PDB: 4C3I) to the final EM densities and manually built regions in which there were significant deviations (mainly around the active site). Built models were real-space refined, yielding complex structures with excellent stereochemistry (Figure S1H; Table 1). The Pol I molecules in EC1 and EC2 show a very similar overall conformation (root-mean-square deviation [RMSD] = 0.93 $Å_{4,124\ C\alpha\ atoms\ aligned}$), despite the different transcription scaffolds. In the EC1, the downstream DNA duplex and the DNA/RNA hybrid are very well defined, with density similar to crystal structures at similar resolution (Figure 2A) (Kettenberger et al., 2004; Westover et al., 2004b). In addition, the complete upstream DNA duplex is visible, while densities for the transcription scaffolds of the OC and EC2 are of lesser quality. The binary OC corresponds to a DNA-bound conformation prior to RNA synthesis or, alternatively, to a conformation where the RNA has been cleaved due to Pol I's intrinsic RNase activity

Table 1. Refinement Statistics

	OC	EC1	EC2	EC_tWH
Model Composition				
No. of chains	14 + 2	14 + 3	14 + 3	14 + 3
Non-hydrogen atoms	34,779	34,490	34,103	35,860
Protein residues	4,271	4,135	4,161	4,383
Nucleic acids	43	79	51	51
Ligand (Zn^{2+})	7	6	6	6
Refinement				
PDB ID	PDB: 5M5W	PDB: 5M5X	PDB: 5M5Y	PDB: 5M64
Resolution (Å)	237.6–3.8	237.6–4.0	237.6–4.0	237.6–4.6
Map sharpening B-factor ($Å^2$)	−109	−108	−89	−124
MolProbity score	2.18	2.21	2.25	2.27
Clash score (all atoms)	11.24	13.09	12	11.91
Rotamer outliers (%)	2.51	1.88	2.3	2.85
Ramachandran statistics: favored (%)	95.61	94.46	94.40	95.21
Ramachandran statistics: disallowed (%)	0.02	0.02	0.07	0.02
RMS (bonds, Å)	0.0033	0.0033	0.0034	0.0034
RMS (angles, °)	0.93	0.92	0.94	0.94
Nucleic acids (RNA): correct sugar puckers (%)	–	92.3	100.0	100.0
Nucleic acids (RNA): good backbone conform (%)	–	77.0	88.4	88.4

(see discussion below). In contrast, EC1/2 and EC_tWH correspond to mature, transcriptionally active, post-translocated elongation complexes.

Compared to the crystal structure of apo Pol I, we observe major conformational changes upon binding of Pol I to the two different transcription scaffolds (Figures 1A and 1B). The structural rearrangements can be grouped into the concerted movement of the two previously characterized modules 1 and 2 (Fernández-Tornero et al., 2013). Module 1 comprises the major part of A190 (excluding the pore 1, funnel, and jaw domains), the C terminus of A135, Rpb5, Rpb6, Rpb8, and the stalk, while module 2 is formed by the rest of A135; the pore 1, funnel, and jaw domains of subunit A190; AC40-AC19; Rpb10; Rpb12; A12.2; and the A49-A34.5 heterodimer. In the OC, module 1 moves toward module 2, narrowing the DNA-binding cleft from 42 to 38 Å. Further closing of the cleft is observed in EC1/2/tWH, which, coupled to the movement of module 2 toward the DNA, narrows the cleft to 31 Å (Figure 1C). In both OC and EC1/2/tWH structures, the progressive closing of the clamp enforces the narrowing of the DNA-binding cleft. The overall reduction in the cleft width between the Pol I dimer observed in the crystal (Engel et al., 2013; Fernández-Tornero et al., 2013) and in solution (Pilsl et al., 2016) and the Pol I EC is 11 Å. Interestingly, this reduction in cleft width is similar to that observed in the monomeric apo Pol I structure in solution (and in the Pol I-Rrn3 complex) compared to the apo Pol I dimer (Pilsl et al., 2016). This suggests that there is only limited further closing of the monomeric apo Pol I upon formation of the Pol I EC. In contrast, the width of the DNA-binding cleft in the fully committed Pol I EC (31 Å) is more similar to the Pol II EC (33 Å) than to Pol III (19 Å) (Hoffmann et al., 2015; Kettenberger et al., 2004). Both Pol I and II mainly synthesize transcripts with a length of above 1 kb, whereas Pol III transcripts have an average size of 100 nt (Jackson et al., 2000). The difference in the cleft width among the eukaryotic RNA polymerases could reflect an adaptation toward their transcription product lengths, which are more similar in Pol I and Pol II than in Pol III. In contrast, narrowing of the DNA-binding cleft and clamp closing appear to be conserved mechanisms in all eukaryotic RNA polymerases.

In all elongating Pol I complexes, the stalk subunits (A43-A14) follow the same movement as the clamp (Figure S3A), which confirms the close coupling between the stalk and the core enzyme, also observed in Pol III (Hoffmann et al., 2015). Accordingly, the relative position of the clamp and stalk in EC1/2 is the same as in the OC (Figure S3B). We observe relatively weak EM density for the distal end (A43 OB domain, residues 128–251) of the stalk in all maps, although it is better resolved in the OC (Figure S3C). In addition, the C-terminal helix of A43, which mediates Pol I dimerization, is also disordered and absent from the reconstructions. This helix is also flexible in the apo Pol I monomer and Rrn3-bound conformations (Engel et al., 2016; Pilsl et al., 2016), supporting its regulatory role during Pol I dimerization.

In the EC1, we also observe helical density above the clamp that we tentatively assign to the N-terminal helix of subunit A43 (Figure 1). This helix is connected to the tip domain (residues 31–128) by a 16-residue loop, which confers independent mobility. In contrast, in the OC, EC2, and EC_tWH, this helix is positioned much closer to the stalk, suggesting that it can adopt different positions during elongation.

Clamp Head and Protrusion Domains Stabilize Downstream and Upstream DNA

In both the OC and EC Pol I structures, the entire downstream DNA is visible and adopts a position in the DNA-binding cleft

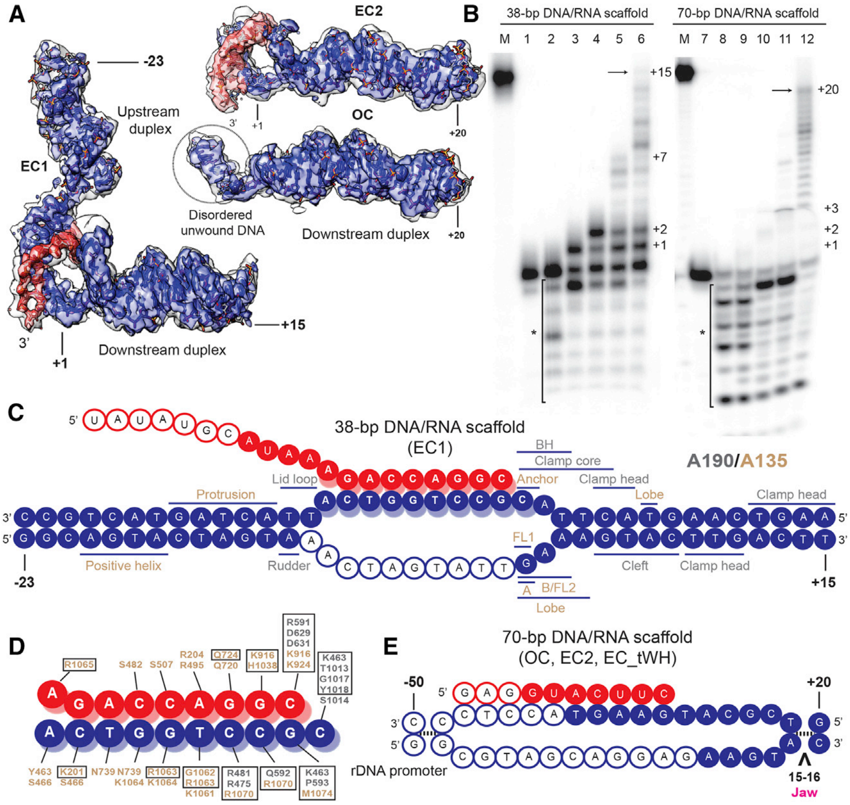

Figure 2. Pol I Interactions with Nucleic Acids

(A) DNA (blue)/RNA (red) densities for the EC1, EC2, and OC. In the OC, no density for the RNA is present. The density for the nucleic acids low-pass filtered at 5 Å resolution is shown in white.

(B) RNA extension assays for the 38-bp and 70-bp DNA/RNA scaffolds. The RNA was radiolabeled at the 5′ end with ^{32}P. Lanes: M, 36-nt RNA marker; 1 and 7, DNA/RNA scaffold minus Pol I; 2 and 8, + Pol I without NTPs; 3 and 9, +Pol I and GTP (38 bp) or ATP (70 bp); 4 and 10; +Pol I and GTP and UTP (38 bp) or ATP and UTP (70 bp); 5 and 11; +Pol I and GTP, UTP, and ATP; and 6 and 12, +Pol I and four NTPs. The positions on the template strand are shown on the right. The asterisk indicates RNA cleavage products.

(C) Schematic representation of the 38-bp DNA/RNA scaffold and Pol I elements that are within 8 Å of the DNA duplexes, colored according to the subunit. Colored circles represent modeled nucleotides, while unfilled ones were not modeled; “A” and “B” refer to loops A and B, respectively.

(D) Residues that are within 5 Å of the template and RNA strands in the DNA/RNA hybrid, colored as in (C). Boxed amino acid residues are identical within Pol I, II, and III.

(E) Schematic representation of the 70-bp DNA/RNA scaffold, as in (C).

See also Figure S4.

similar to other RNA polymerases (Figures 1 and 2A). Notably, the clamp head module of A190 lies in close proximity to the downstream end, causing an asymmetry in the position of the duplex in the cleft as in Pol II and Pol III (Barnes et al., 2015; Hoffmann et al., 2015). The Rpb5 jaw, which mediates downstream DNA binding of Pol II and Pol III, is also in a conserved position, suggesting that contacts in this region are maintained in all three eukaryotic RNA polymerases. However, compared to EC1/2, the downstream duplex in the OC is tilted toward the interface between the Rpb5 jaw and the clamp head (Figure S4A, right panel), probably because the cleft is not yet completely closed. Interestingly, we observe no RNA density in the OC (Figure 2A), as was previously reported for a Pol II open complex (He et al., 2016). In both cases, the catalytic domain of the conserved transcription factor II S (TFIIS)-like factor (TFIIS in Pol II and A12.2 in Pol I) is present in the active site, while it is absent in the EC1/2/tWH structures. The presence of the catalytic domain A12.2C might affect the stability of the DNA/RNA hybrid and/or induce backtracking and A12.2-mediated RNA cleavage (Figure 2B). However, a similar binary OC has also been observed in Pol II in the absence of TFIIS-like factors (Cheung et al., 2011), suggesting that Pol I is also able to bind and position DNA in the absence of RNA.

Strikingly, we observe density for the upstream DNA duplex in EC1, but not in OC and EC2 (Figure 2A). This is rather unexpected, for upstream DNA is not visible in the EC of Pol II and Pol III using a similar transcription bubble (Hoffmann et al., 2015; Kettenberger et al., 2004). Nevertheless, upstream DNA density has been observed in cryo-EM reconstructions of mammalian Pol II with focused classifications (Bernecky et al., 2016), and in the crystal structure of Pol II, where, in presence of TFIIF, longer DNA was used for co-crystallization (Barnes et al., 2015). The complete closing of the cleft by the movement of modules 1 and 2 toward each other positions part of the protrusion domain of A135 (residues 405–470), including several positively charged residues, close to the upstream DNA (Figures 2C and S4A, left panel). In particular, basic residues R434, K441, K443, R444, R448, and R452 reach toward the phosphate backbone of the DNA duplex around positions −17 to −20 (position −1 corresponds to the annealed 3′ end of the RNA). The loop connecting the two DNA-contacting helices (“helix A” and “positive helix” in Figure S4) contains two (Pol I and III) or one (Pol II) positively charged residue, while the “positive helix” is not conserved between yeast Pol I, Pol II, and Pol III (Figure S4B). Pol II establishes contacts with the upstream DNA through the Rpb2 “wedge” (residues 862–874) (Barnes et al., 2015). However, in all elongating Pol I complexes the corresponding loop (A135, residues 813–819) is disordered, although it contains a glycine, as in Pol II (G818), and two arginines that could potentially interact with the phosphate backbone. Interestingly, the upstream DNA in the Pol I EC1 is slightly shifted toward the protrusion, apparently favoring the interaction with the “positive helix,” while in Pol II the DNA duplex is positioned closer to the “wedge” (Figure S4A, left panel). This short helix could promote a tighter binding with the upstream DNA, thereby increasing the stability of the transcribing

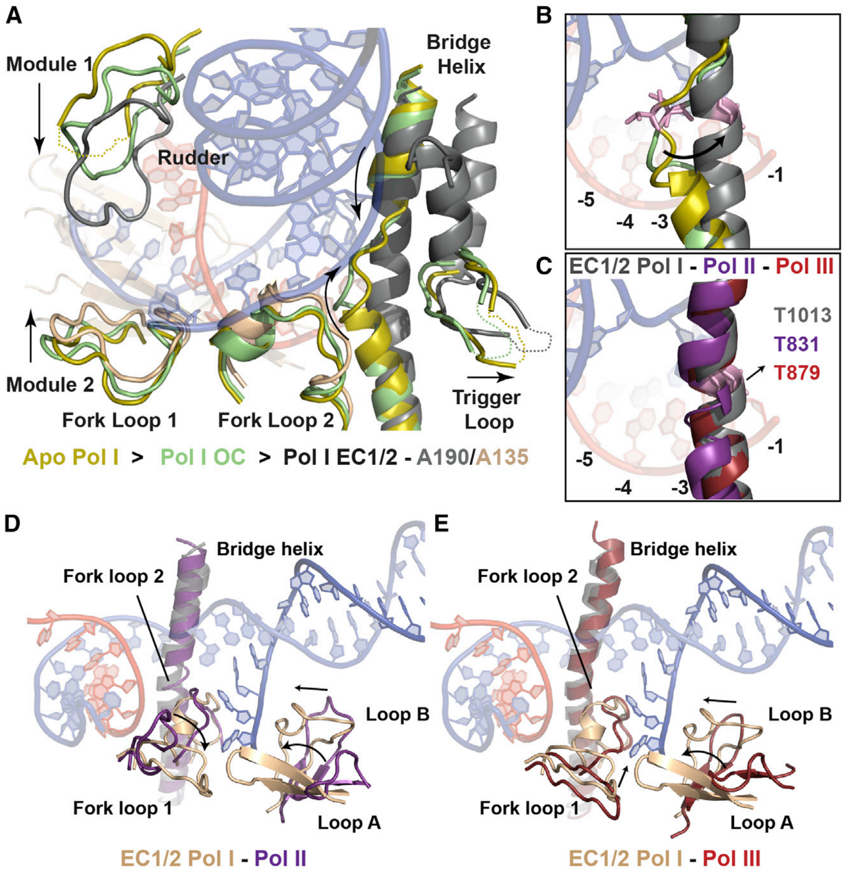

Figure 3. Pol I Active Site Elements and Its Comparison with Pol II and III

(A) Functional elements in the active site are shown for apo Pol I (yellow), OC Pol I (green), and EC1/2 Pol I (dark gray/wheat). The BH gradually folds upon cleft closure together with movements of modules 1 and 2.

(B) Folding of the BH repositions the conserved threonine 1013 (pink) close to the RNA at position −1.

(C) Threonine 1013 is a conserved residue in Pol II (purple) and Pol III (red).

(D and E) The FL1 and loops A and B in the active site of Pol I form a narrow passage that presumably directs the path of the NT DNA strand. Pol II (purple; D) and Pol III (red; E) don't show similar arrangements of these elements.

complex, while absence of upstream DNA density in the EC2 could be due to the longer and presumably more flexible upstream DNA duplex.

Active Site Changes during the Transition to the Elongation Complex

As in Pol II and Pol III, the DNA duplex in the EC1 is unwound from position +2 in the downstream end to position −11. Although most active site elements adopt similar conformations in apo Pol I, OC, and EC1/2, they move in a concerted manner and include a network of loops that may stabilize the open bubble, as discussed below (Figures 2C–2E and 3A). In contrast, we observe different conformations of the middle region of the BH. Compared to apo Pol I (Engel et al., 2013; Fernández-Tornero et al., 2013), the unwound region of the BH (A190 residues 1012–1016) progressively adopts a helical conformation during the transition to elongation (Figure 3A). While this region is well resolved in EC1/2, it shows weaker density in the OC and is disordered in the cryo-EM structures of monomeric apo Pol and its initiation-competent form (Engel et al., 2016; Pilsl et al., 2016), suggesting that RNA synthesis, rather than closing of the DNA-binding cleft, triggers the complete folding of the BH. Accordingly, this mobile region includes a conserved threonine A190 T1013 (T831 in Pol II-Rpb1 and T879 in Pol III-C160), which has been proposed as a "probe" for DNA/RNA stability and to be important for TFIIS-stimulated RNA cleavage (Da et al., 2016). In the OC, residue T1013 is in a similar position as in apo Pol I, facing away from base position +1. Folding of the BH shifts its position toward the 3′ end of the RNA (Figure 3B). Remarkably, the position of this residue is the same in the ECs of all three Pols, suggesting a conserved mechanism (Figure 3C) (Hoffmann et al., 2015; Kettenberger et al., 2004; Westover et al., 2004a). Folding and relaxation of the BH during translocation has been proposed in bacterial RNA polymerase and in Pol II (reviewed by Cheung and Cramer, 2012). The observation of different states of the BH in DNA-bound Pol I complexes suggests that folding and relaxation of the BH might also occur in Pol I.

Although the tip of the trigger loop (TL) is disordered in the OC and EC1/2, it adopts an "open" conformation (reviewed in Martinez-Rucobo and Cramer, 2013). Accordingly, conserved residues previously involved in interactions with the nucleoside triphosphate (NTP) and other residues in the BH are in the same conformation as in the post-translocated Pol II and Pol III EC (Hoffmann et al., 2015; Kettenberger et al., 2004; Wang et al., 2006). This suggests that after translocation, Pol I, II, and III adopt similar conformations. In Pol II, the upstream DNA template strand is separated from the RNA through conserved elements, namely the rudder, the lid, and fork loop 1 (FL1) (Westover et al., 2004b). In Pol I, the rudder (A190, residues 443–455), although flexible, becomes more ordered in the OC and EC1/2 compared to apo Pol I, and its position roughly overlaps with Pol II and Pol III but points toward the lobe instead of the protrusion. In contrast, the lid loop is not well resolved in the Pol I complexes compared to apo Pol I, which is different from the situation in Pol II and Pol III (Gnatt et al., 2001; Hoffmann et al., 2015; Kettenberger et al., 2004). FL1 (A135 residues 470–484) and FL2 (A135 residues 505–520) are both implicated in stabilizing the transcription bubble and preventing DNA re-association. Both loops adopt similar positions as in apo Pol I. However, comparison with Pol II and Pol III reveals a different conformation of FL1 (Figures 3D and 3E). As in Pol III, the FL1

is in an open conformation, but is tilted toward the non-template (NT) strand. Notably, it forms a barrier with a β-hairpin (loop A) of the lobe (A135 residues 260– 271) that would clash with the NT strand in the Pol II EC (Figure 3D). In Pol II and Pol III, this extension is shorter and differently positioned. In contrast, FL2 adopts a position similar to Pol II and Pol III, sterically blocking duplex formation at the downstream end of the bubble. Interestingly, we observe another element from the lobe moving up toward the NT strand (residues 218–232, loop B) and narrowing the path for the NT single-strand region. The interplay among these four elements (FL1, FL2, loop A, and loop B) apparently stabilizes the open transcription bubble by interacting with the NT strand. Moreover, the position of loops A and B forces a different direction for the NT strand than in Pol II, which could influence the catalytic properties of the enzyme. Finally, DNA unwinding and interactions with the template DNA strand seem to be similar as in Pol II and Pol III, and are mediated by different elements of subunits A190 and A135 (Figures 2C and 2D).

In the crystal structure of apo Pol I, the "DNA-mimicking loop" (A190 residues 1340–1400) was shown to interact with a Pol I-specific arginine (A190 R1015) in the unwound region of the BH and with the aspartate loop, thereby stabilizing an inactive Pol I conformation and regulating its activity (Engel et al., 2013; Fernández-Tornero et al., 2013). Consistent with its incompatibility in transcription, we do not observe any density corresponding to the DNA-mimicking loop in the OC or EC1/2 within the DNA-binding cleft or at any other region of Pol I. Moreover, folding of the BH changes the position of A190 R1015 and presumably contributes to destabilizing the interaction of the DNA-mimicking loop with the DNA-binding cleft.

A49 tWH and A12.2 C-Terminal Domains Are Mobile Elements

The A49-A34.5 heterodimer associates to the lobe of Pol I and anchors the N-terminal domain of subunit A12.2 (Fernández-Tornero et al., 2013). The heterodimer subunit A34.5 connects with a long C-terminal linker to a distant anchor site, promoting a permanent association with the core, while the C-terminal A49 tWH might act as a functional counterpart of TFIIE in Pol I (Vannini and Cramer, 2012). In the OC and the EC1/2 structures, binding of the heterodimer β-barrel to the core is conserved compared to the apo Pol I structure. Interestingly, the density corresponding to the A49-A34.5 heterodimer is stronger in the OC than in EC1/2, which suggests that this module becomes more flexible during elongation. The A49 tWH has been shown to be important for processivity both in vitro and in vivo (Beckouet et al., 2008; Geiger et al., 2010; Pilsl et al., 2016), and previous studies have placed this flexible domain near the DNA-binding cleft (Jennebach et al., 2012; Pilsl et al., 2016). We fitted the crystal structure of the A49 tWH (PDB: 3NFI) in extra density that appeared in the EC_tWH reconstruction in the interface between the stalk and the upstream DNA (Figure 4A). Notably, the WH2 domain and the C-terminal tail, shown to be important for DNA binding in vitro (Geiger et al., 2010), point toward the upstream DNA (Figure 4B). Moreover, residues 367–415, which, when deleted, cause a cold-sensitive phenotype (Beckouet et al., 2008), lie next to the DNA. Additional density, presumably corresponding to the A49 linker, spans over the DNA-binding cleft toward the A49 dimerization domain, while the C-terminal tail (403–415) is disordered. The position of A49 tWH matches the proposed functional and structural relationship with TFIIE (Hoffmann et al., 2016; Plaschka et al., 2016; Vannini and Cramer, 2012). Binding of the tWH to the upstream DNA could stabilize its interaction with the protrusion domain and enforce a closed clamp, thereby enhancing processivity.

The A49-A34.5 heterodimer also has a stimulatory effect on the RNA cleavage activity of the A12.2 C-terminal domain (A12.2C) (Geiger et al., 2010). A12.2C is homologous to the catalytic domain III of TFIIS and in the apo Pol I crystal structure adopts a similar position in the DNA-binding cleft, close to the NTP entry pore and the funnel domain (Cheung and Cramer, 2011; Engel et al., 2013; Fernández-Tornero et al., 2013), while A12.2C is absent from monomeric Pol I (Pilsl et al., 2016). In the reconstruction of the OC, density for the entire A12.2 subunit, including A12.2C, is also present (Figure 4C), while in EC1/2 and EC_tWH the N-terminal and linker regions are visible, but density for A12.2C is absent (Figure 4D). Although the position of the A12.2C in the OC is similar to that in apo Pol I, the catalytic loop appears to be flexible, as also observed in the Pol I-Rrn3 complex (Engel et al., 2016). Conceivably, the partial cleft closing and the presence of the A12.2C in the active site in the OC resemble an intermediate state where Pol I can either move forward toward promoter escape and productive initiation or toward abortive initiation (Conaway et al., 2000). Consequently, the establishment of extensive contacts with the RNA and the upstream DNA, as well as further clamp closing in the EC, might displace A12.2C from the active site. Alternatively, it is also possible that in this conformation, the A12.2C is stabilized for early backtracking events. The position of A12.2C in the OC is similar to the position of TFIIS domain III in an arrested-reactivation intermediate of Pol II (Cheung and Cramer, 2011), suggesting that it might represent a complex in which A12.2C has cleaved the RNA. Accordingly, the intrinsic RNA cleavage activity of Pol I is stronger with the 70-bp compared to the 38-bp transcription scaffold (Figure 2B), which we attribute to differences in RNA length and transcription scaffold design. It has also been hypothesized that A12.2C interferes with one-dimensional diffusion of the enzyme along the DNA during pausing, thereby preventing the formation of large-scale backtracking (Lisica et al., 2016). Conversely, displacement of A12.2C would increase diffusion and likely the processivity of the enzyme. Importantly, the absence of a C-terminal TFIIS-like domain in the cleft during transcription elongation is a conserved trait among all eukaryotic RNA polymerases (Hoffmann et al., 2015) that is now also observed in Pol I.

Conclusions and Perspective

The cryo-EM structures of elongating Pol I further increase our understanding of the Pol I transcription cycle (Figure 4E). Upon binding to DNA, the Pol I DNA-binding cleft progressively closes and the BH folds. The different BH conformation in the OC and EC1/2 suggests that the BH can adopt different conformations in the presence of nucleic acids, in line with current models of translocation (Bar-Nahum et al., 2005; Silva et al., 2014). In contrast to the pronounced changes in the BH, most other active site elements adopt similar conformations as in apo Pol I,

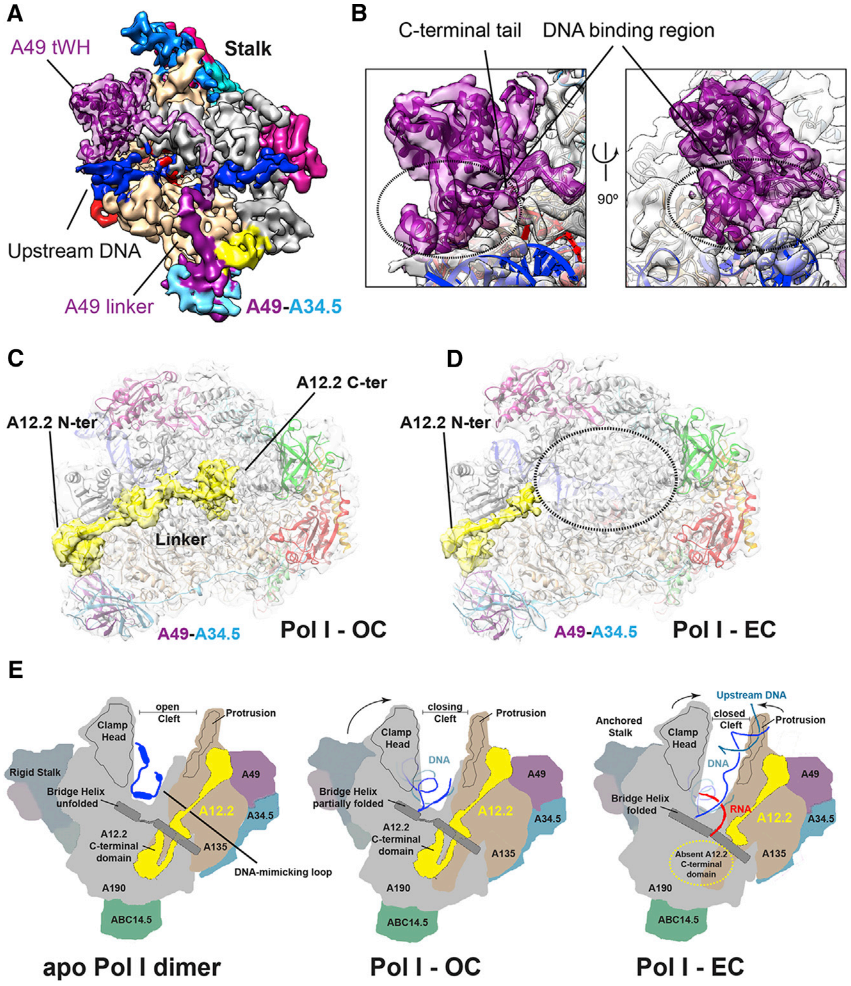

Figure 4. Position of the A49 tWH, Displacement of the A12.2 C-Terminal Zn^{2+} Ribbon from the Cleft, and Pol I Transition upon DNA Binding

(A) Cryo-EM map of the EC_tWH low-pass filtered to 8 Å resolution and colored according to the subunits as in Figure 1. The density corresponding to the A49 tWH is shown in transparency with the crystal structure (PDB: 3NFI) fitted. The proposed connection between the A49 dimerization domain and the tWH is colored like A49.

(B) Close-up views of the fitted density showing the position of the DNA-binding region (circle) and the position of the C-terminal tail.

(C and D) A12.2 density (shown in yellow) in the Pol I-OC (C) is displaced in the Pol I-EC (D). A12.2 density is filtered to 5 Å resolution for better visualization. The dotted black circle in the Pol I-EC demonstrates the absence of additional density, as opposed to the Pol I-OC, where the A12.2 C-terminal domain is visible.

(E) Schematic illustration of apo Pol I dimer (left), Pol I-OC (middle), and Pol I-EC (right) in back view. Subunit A12.2 and the BH are drawn separately; clamp head and protrusion domains are indicated by an encircled line. The DNA-mimicking loop is depicted in blue in apo Pol I.

although the movement of modules 1 and 2 brings these elements closer to the DNA/RNA hybrid. In particular, two loops protruding from the A135 lobe domain are in close proximity to the NT strand and could change the path of the NT strand through the cleft. After forming a stable DNA/RNA hybrid, the clamp further closes, thereby narrowing the DNA-binding cleft, while movement of module 2 brings fork loops, lobe, and protrusion domain into close contact with the transcription bubble. These conformational changes further stabilize the DNA/RNA hybrid in a mature EC, but also displace A12.2C from the DNA-binding cleft. In conclusion, Pol I uses conserved functional mechanisms, but also Pol I-specific features, for transcription elongation. The functional roles of these specific features now need to be further explored using complementary genetic and biochemical approaches.

EXPERIMENTAL PROCEDURES

Protein Purification and Complex Assembly

Pol I was purified from *S. cerevisiae* using an AC40 TAP-tag purification protocol as previously described (Moreno-Morcillo et al., 2014), except that purified Pol I was exchanged for EM buffer (150 mM $(NH_4)_2SO_4$, 15 mM HEPES-NaOH [pH 7.5], and 10 mM DTT). For the EC1, Pol I was mixed with a 5-fold molar excess of a pre-annealed 38-bp transcription scaffold with an 11-nt mismatch region as previously described (Hoffmann et al., 2015), except that a 20-nt RNA (5′-UAUAUGCAUAAAGACCAGGC-3′) was used. Briefly, the template (T) and non-template (NT) strands were mixed at a final concentration of 50 μM and annealed by heating to 95°C in RNase-free water, and then slowly cooled to 25°C in 1 hr. Then, an equimolar amount of RNA was added and annealed by heating the sample to 45°C, then cooled down to 4°C. The complex was incubated for 1 hr at 4°C to a final concentration of 1 mg/mL in EM buffer. For the OC, EC2, and EC_tWH, Pol I was incubated with a pre-annealed 70-bp transcription scaffold (prepared as described above) containing the core promoter sequence (−50 to +20) (T, 5′-GTCTTCAACTGCTTTCGCATGAAGTACCTCCCAACTACTTTTCCTCACACTTGTACTCCATGACTAAACC-3′; NT, 5′-GGTTTAGTCATGGAGTACAAGTGTGAGGAAAAGT AGTTGGCGTAGCAGGAGAAGTAAAGCAGTTGAAGAC-3′) and a 15-nt mismatch region with a 10-nt RNA (5′-GAGGUACUUC-3′) in 100 mM potassium acetate, 50 mM HEPES-NaOH (pH 7.5), 5 mM magnesium acetate, and 10 mM DTT. Both mismatch-containing scaffolds are artificial and may differ from an in vivo-created, fully complementary transcription bubble. The sample also contained Pol I-specific transcription factors Rrn3 and core factor, but only a minor fraction of particles contained density corresponding to these proteins.

Sample Preparation

Samples were diluted to 0.2 mg/mL and immediately used for grid preparation. A total of 2.5 μL of sample was applied on freshly glow-discharged Quantifoil grids (400 mesh holey carbon 1.2/1.3 molybdenum for EC1 and 200 mesh holey carbon 2/1 copper for OC, EC2, and EC_tWH) in an FEI Vitrobot Mark II at 20°C and 100% humidity. The sample was incubated for 15 s, blotted for 8 s, and flash frozen in liquid ethane.

Electron Microscopy

Data were acquired on FEI Titan Krios operating at 300 keV through a Gatan Quantum 967 LS energy filter using a 20 eV slit width in zero-loss mode. Movie frames were recorded on a Gatan K2-Summit direct electron detector at a

nominal EFTEM (energy-filtered transmission electron microscope) magnification of 105,000× corresponding to 1.35 Å calibrated pixel size (in 4K mode). A total of 715 and 4,235 movie frames were collected for EC1 and OC/EC2/EC_tWH, respectively, using a defocus range of −0.75 to −4 μm. For both datasets, 20 super-resolution frames were collected with a dose rate of 2 e^{-} $Å^{-2}$ s^{-1} for a total dose of 40 e^{-} $Å^{-2}$. Data collection was fully automated using SerialEM (Mastronarde, 2005).

Image Processing and Model Building

Acquired cryo-EM images were processed using RELION-1.4 (Scheres, 2012), and models were built using COOT (Emsley and Cowtan, 2004) and UCSF Chimera (Pettersen et al., 2004). Figures were prepared using Chimera and PyMol (Schrodinger, 2010). Further details can be found in the Supplemental Experimental Procedures.

RNA Extension Assay

The 20-nt and 10-nt RNA oligonucleotides were radiolabeled with ^{32}P by T4 PNK and gel purified on denaturing 15% urea-PAGE, for reactions using either the 38-bp or the 70-bp transcription scaffold. A total of 2 pmol of pre-annealed transcription scaffold was incubated with 4 pmol Pol I for 20 min at 20°C in EM buffer in the presence of 5 mM $MgCl_2$, and the reaction was initiated by adding the corresponding NTP(s) at a final concentration of 250 μM. RNA extension was performed at 28°C for 20 min. The reaction was stopped by adding loading buffer (8 M urea, TBE) and heating for 2 min at 95°C. The resulting RNA product was analyzed on a denaturing polyacrylamide gel (17% PAGE, 8 M urea) using an FLA7000 phosphoimager (Fujifilm).

ACCESSION NUMBERS

The accession numbers for the cryo-EM maps are Electron Microscopy Data Bank (EMD): EMD-3446 (Pol I OC), EMD-3447 (Pol I EC1), EMD-3448 (Pol I EC2), and EMD-3449 (Pol I EC_tWH). The accession numbers for the coordinates of the corresponding atomic models are PDB: 5M5W (Pol I OC), 5M5X (Pol I EC1), 5M5Y (Pol I EC2), and 5M64 (Pol I EC_tWH).

SUPPLEMENTAL INFORMATION

Supplemental Information includes Supplemental Experimental Procedures and four figures and can be found with this article online at http://dx.doi.org/10.1016/j.molcel.2016.11.013.

AUTHOR CONTRIBUTIONS

C.W.M. initiated and supervised the project. L.T. and Y.S. established the sample preparation and grid freezing conditions. L.T., Y.S., and W.J.H.H. collected cryo-EM data. L.T. and N.A.H. built the initial models. A.J.J. and C.S. implemented the refinement protocol. A.J.J., N.A.H., and L.T. revised the models and optimized the refinement protocol. R.W. was responsible for the fermentation of Pol I. L.T., N.A.H., Y.S., and C.W.M. wrote the manuscript with the input of the other authors.

ACKNOWLEDGMENTS

L.T., Y.S., R.W., and C.W.M. acknowledge support by the ERC Advanced Grant (ERC-2013-AdG340964-POL1PIC). N.A.H. and A.J.J. acknowledge support by the EMBL International PhD program and the EMBL Interdisciplinary Postdoc Program (EIPOD) under Marie Curie COFUND actions (PCOFUND-GA-2008-229597), respectively.

Received: August 2, 2016
Revised: September 26, 2016
Accepted: November 4, 2016
Published: November 17, 2016

REFERENCES

Bar-Nahum, G., Epshtein, V., Ruckenstein, A.E., Rafikov, R., Mustaev, A., and Nudler, E. (2005). A ratchet mechanism of transcription elongation and its control. Cell *120*, 183–193.

Barnes, C.O., Calero, M., Malik, I., Graham, B.W., Spahr, H., Lin, G., Cohen, A.E., Brown, I.S., Zhang, Q., Pullara, F., et al. (2015). Crystal structure of a transcribing RNA polymerase II complex reveals a complete transcription bubble. Mol. Cell *59*, 258–269.

Beckouet, F., Labarre-Mariotte, S., Albert, B., Imazawa, Y., Werner, M., Gadal, O., Nogi, Y., and Thuriaux, P. (2008). Two RNA polymerase I subunits control the binding and release of Rrn3 during transcription. Mol. Cell. Biol. *28*, 1596–1605.

Bernecky, C., Herzog, F., Baumeister, W., Plitzko, J.M., and Cramer, P. (2016). Structure of transcribing mammalian RNA polymerase II. Nature *529*, 551–554.

Bischler, N., Brino, L., Carles, C., Riva, M., Tschochner, H., Mallouh, V., and Schultz, P. (2002). Localization of the yeast RNA polymerase I-specific subunits. EMBO J. *21*, 4136–4144.

Brueckner, F., and Cramer, P. (2008). Structural basis of transcription inhibition by alpha-amanitin and implications for RNA polymerase II translocation. Nat. Struct. Mol. Biol. *15*, 811–818.

Cheung, A.C., and Cramer, P. (2011). Structural basis of RNA polymerase II backtracking, arrest and reactivation. Nature *471*, 249–253.

Cheung, A.C., and Cramer, P. (2012). A movie of RNA polymerase II transcription. Cell *149*, 1431–1437.

Cheung, A.C., Sainsbury, S., and Cramer, P. (2011). Structural basis of initial RNA polymerase II transcription. EMBO J. *30*, 4755–4763.

Colis, L., Peltonen, K., Sirajuddin, P., Liu, H., Sanders, S., Ernst, G., Barrow, J.C., and Laiho, M. (2014). DNA intercalator BMH-21 inhibits RNA polymerase I independent of DNA damage response. Oncotarget *5*, 4361–4369.

Conaway, J.W., Shilatifard, A., Dvir, A., and Conaway, R.C. (2000). Control of elongation by RNA polymerase II. Trends Biochem. Sci. *25*, 375–380.

Da, L.T., Pardo-Avila, F., Xu, L., Silva, D.A., Zhang, L., Gao, X., Wang, D., and Huang, X. (2016). Bridge helix bending promotes RNA polymerase II backtracking through a critical and conserved threonine residue. Nat. Commun. *7*, 11244.

Drygin, D., Rice, W.G., and Grummt, I. (2010). The RNA polymerase I transcription machinery: an emerging target for the treatment of cancer. Annu. Rev. Pharmacol. Toxicol. *50*, 131–156.

Drygin, D., Lin, A., Bliesath, J., Ho, C.B., O'Brien, S.E., Proffitt, C., Omori, M., Haddach, M., Schwaebe, M.K., Siddiqui-Jain, A., et al. (2011). Targeting RNA polymerase I with an oral small molecule CX-5461 inhibits ribosomal RNA synthesis and solid tumor growth. Cancer Res. *71*, 1418–1430.

Emsley, P., and Cowtan, K. (2004). Coot: model-building tools for molecular graphics. Acta Crystallogr. D Biol. Crystallogr. *60*, 2126–2132.

Engel, C., Sainsbury, S., Cheung, A.C., Kostrewa, D., and Cramer, P. (2013). RNA polymerase I structure and transcription regulation. Nature *502*, 650–655.

Engel, C., Plitzko, J., and Cramer, P. (2016). RNA polymerase I-Rrn3 complex at 4.8 Å resolution. Nat. Commun. *7*, 12129.

Fernández-Tornero, C., Moreno-Morcillo, M., Rashid, U.J., Taylor, N.M., Ruiz, F.M., Gruene, T., Legrand, P., Steuerwald, U., and Müller, C.W. (2013). Crystal structure of the 14-subunit RNA polymerase I. Nature *502*, 644–649.

Geiger, S.R., Lorenzen, K., Schreieck, A., Hanecker, P., Kostrewa, D., Heck, A.J., and Cramer, P. (2010). RNA polymerase I contains a TFIIF-related DNA-binding subcomplex. Mol. Cell *39*, 583–594.

Gnatt, A.L., Cramer, P., Fu, J., Bushnell, D.A., and Kornberg, R.D. (2001). Structural basis of transcription: an RNA polymerase II elongation complex at 3.3 A resolution. Science *292*, 1876–1882.

Grummt, I. (2003). Life on a planet of its own: regulation of RNA polymerase I transcription in the nucleolus. Genes Dev. *17*, 1691–1702.

He, Y., Yan, C., Fang, J., Inouye, C., Tjian, R., Ivanov, I., and Nogales, E. (2016). Near-atomic resolution visualization of human transcription promoter opening. Nature *533*, 359–365.

Hoffmann, N.A., Jakobi, A.J., Moreno-Morcillo, M., Glatt, S., Kosinski, J., Hagen, W.J., Sachse, C., and Müller, C.W. (2015). Molecular structures of unbound and transcribing RNA polymerase III. Nature *528*, 231–236.

Hoffmann, N.A., Sadian, Y., Tafur, L., Kosinski, J., and Müller, C.W. (2016). Specialization versus conservation: How Pol I and Pol III use the conserved architecture of the pre-initiation complex for specialized transcription. Transcription *7*, 127–132.

Jackson, D.A., Pombo, A., and Iborra, F. (2000). The balance sheet for transcription: an analysis of nuclear RNA metabolism in mammalian cells. FASEB J. *14*, 242–254.

Jennebach, S., Herzog, F., Aebersold, R., and Cramer, P. (2012). Crosslinking-MS analysis reveals RNA polymerase I domain architecture and basis of rRNA cleavage. Nucleic Acids Res. *40*, 5591–5601.

Kettenberger, H., Armache, K.J., and Cramer, P. (2004). Complete RNA polymerase II elongation complex structure and its interactions with NTP and TFIIS. Mol. Cell *16*, 955–965.

Lisica, A., Engel, C., Jahnel, M., Roldán, É., Galburt, E.A., Cramer, P., and Grill, S.W. (2016). Mechanisms of backtrack recovery by RNA polymerases I and II. Proc. Natl. Acad. Sci. USA *113*, 2946–2951.

Martinez-Rucobo, F.W., and Cramer, P. (2013). Structural basis of transcription elongation. Biochim. Biophys. Acta *1829*, 9–19.

Mastronarde, D.N. (2005). Automated electron microscope tomography using robust prediction of specimen movements. J. Struct. Biol. *152*, 36–51.

Milkereit, P., Schultz, P., and Tschochner, H. (1997). Resolution of RNA polymerase I into dimers and monomers and their function in transcription. Biol. Chem. *378*, 1433–1443.

Moreno-Morcillo, M., Taylor, N.M., Gruene, T., Legrand, P., Rashid, U.J., Ruiz, F.M., Steuerwald, U., Müller, C.W., and Fernández-Tornero, C. (2014). Solving the RNA polymerase I structural puzzle. Acta Crystallogr. D Biol. Crystallogr. *70*, 2570–2582.

Pettersen, E.F., Goddard, T.D., Huang, C.C., Couch, G.S., Greenblatt, D.M., Meng, E.C., and Ferrin, T.E. (2004). UCSF Chimera–a visualization system for exploratory research and analysis. J. Comput. Chem. *25*, 1605–1612.

Pilsl, M., Crucifix, C., Papai, G., Krupp, F., Steinbauer, R., Griesenbeck, J., Milkereit, P., Tschochner, H., and Schultz, P. (2016). Structure of the initiation-competent RNA polymerase I and its implication for transcription. Nat. Commun. *7*, 12126.

Plaschka, C., Hantsche, M., Dienemann, C., Burzinski, C., Plitzko, J., and Cramer, P. (2016). Transcription initiation complex structures elucidate DNA opening. Nature *533*, 353–358.

Poortinga, G., Quinn, L.M., and Hannan, R.D. (2015). Targeting RNA polymerase I to treat MYC-driven cancer. Oncogene *34*, 403–412.

Scheres, S.H. (2012). RELION: implementation of a Bayesian approach to cryo-EM structure determination. J. Struct. Biol. *180*, 519–530.

Schrodinger, L.L.C. (2010). The PyMOL molecular graphics system, version 1.3r1.

Silva, D.A., Weiss, D.R., Pardo Avila, F., Da, L.T., Levitt, M., Wang, D., and Huang, X. (2014). Millisecond dynamics of RNA polymerase II translocation at atomic resolution. Proc. Natl. Acad. Sci. USA *111*, 7665–7670.

Tuske, S., Sarafianos, S.G., Wang, X., Hudson, B., Sineva, E., Mukhopadhyay, J., Birktoft, J.J., Leroy, O., Ismail, S., Clark, A.D., Jr., et al. (2005). Inhibition of bacterial RNA polymerase by streptolydigin: stabilization of a straight-bridge-helix active-center conformation. Cell *122*, 541–552.

Vannini, A., and Cramer, P. (2012). Conservation between the RNA polymerase I, II, and III transcription initiation machineries. Mol. Cell *45*, 439–446.

Wang, D., Bushnell, D.A., Westover, K.D., Kaplan, C.D., and Kornberg, R.D. (2006). Structural basis of transcription: role of the trigger loop in substrate specificity and catalysis. Cell *127*, 941–954.

Warner, J.R. (1999). The economics of ribosome biosynthesis in yeast. Trends Biochem. Sci. *24*, 437–440.

Weinzierl, R.O. (2011). The Bridge Helix of RNA polymerase acts as a central nanomechanical switchboard for coordinating catalysis and substrate movement. Archaea *2011*, 608385.

Westover, K.D., Bushnell, D.A., and Kornberg, R.D. (2004a). Structural basis of transcription: nucleotide selection by rotation in the RNA polymerase II active center. Cell *119*, 481–489.

Westover, K.D., Bushnell, D.A., and Kornberg, R.D. (2004b). Structural basis of transcription: separation of RNA from DNA by RNA polymerase II. Science *303*, 1014–1016.

Molecular Cell, Volume *64*

Supplemental Information

Molecular Structures

of Transcribing RNA Polymerase I

Lucas Tafur, Yashar Sadian, Niklas A. Hoffmann, Arjen J. Jakobi, Rene Wetzel, Wim J.H. Hagen, Carsten Sachse, and Christoph W. Müller

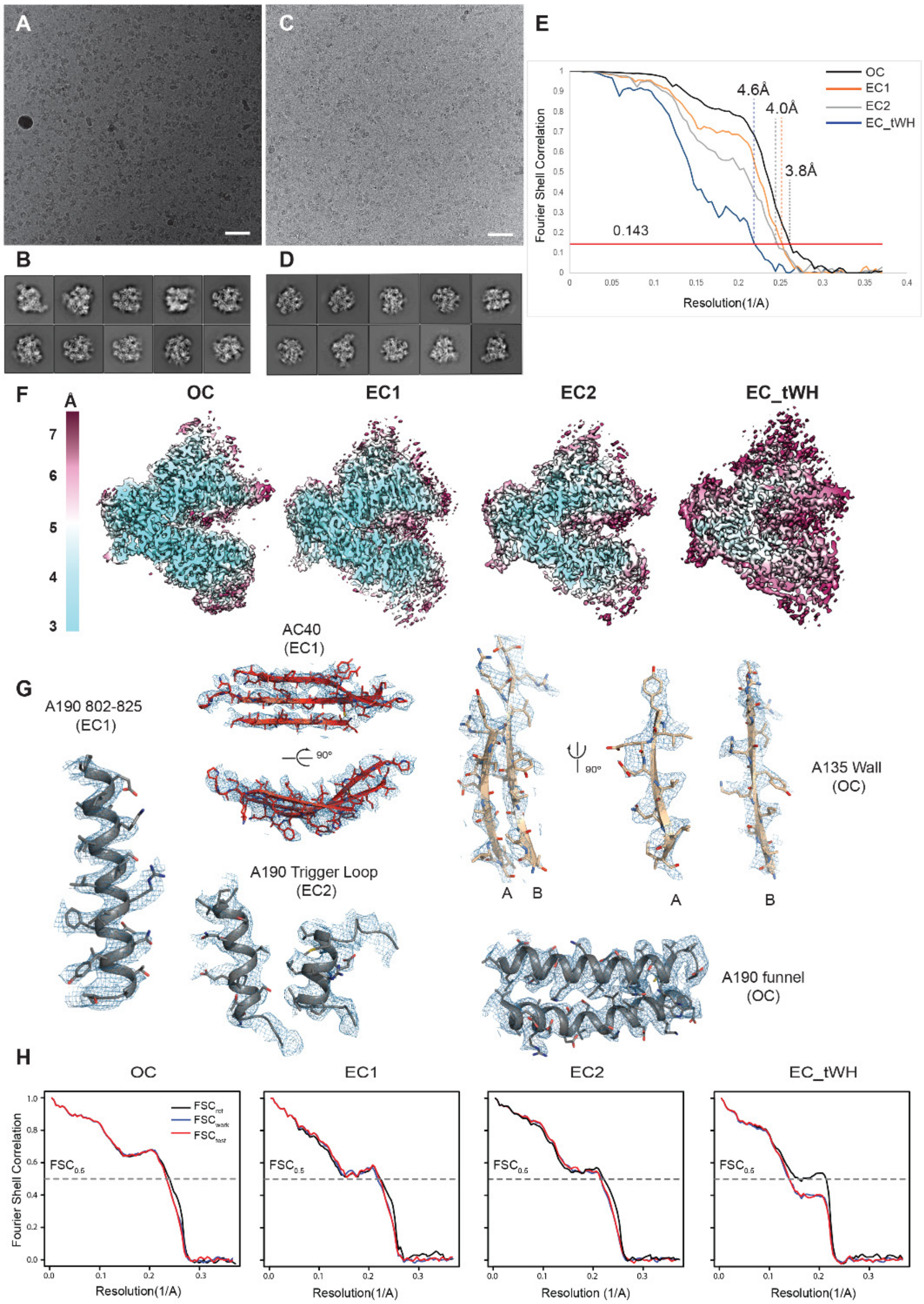

Figure S1. Related to Figure 1. Cryo-EM reconstructions of the Pol I OC, EC1, EC2 and EC_tWH. A, **C**, Representative micrographs for the Pol I OC, EC2, EC_tWH and for the EC1, respectively. Scale bar = 50 nm. **B**, **D**, Representative 2D class averages. **E**, FSC curves of the final reconstructions. The red line indicates the resolution cutoff according to the 0.143 criterion. **F,** The local resolution is displayed on a cross-section of the final electron microscopy maps. **G**, Representative electron microscopy densities of the EC, EC2 and OC. Features such as alpha-helical pitch, large side-chain density and beta-strand separation are discernible **H**, FSC curves calculated between the refined atomic model of OC, EC1, EC2 and EC_tWH, and the half map used in refinement (FSC_{work}) are shown in blue, those calculated between the refined atomic model and the second half map not used for refinement (FSC_{test}) in red. The FSC 0.5 is shown as dashed line. Close agreement between FSC_{work} and FSC_{test} and the absence of a sharp drop beyond the refinement target resolution indicate that no overfitting took place. As reference, the FSC between the refined atomic model and the map obtained from 3D reconstruction using the entire data set (FSC_{ref}) is also shown (black).

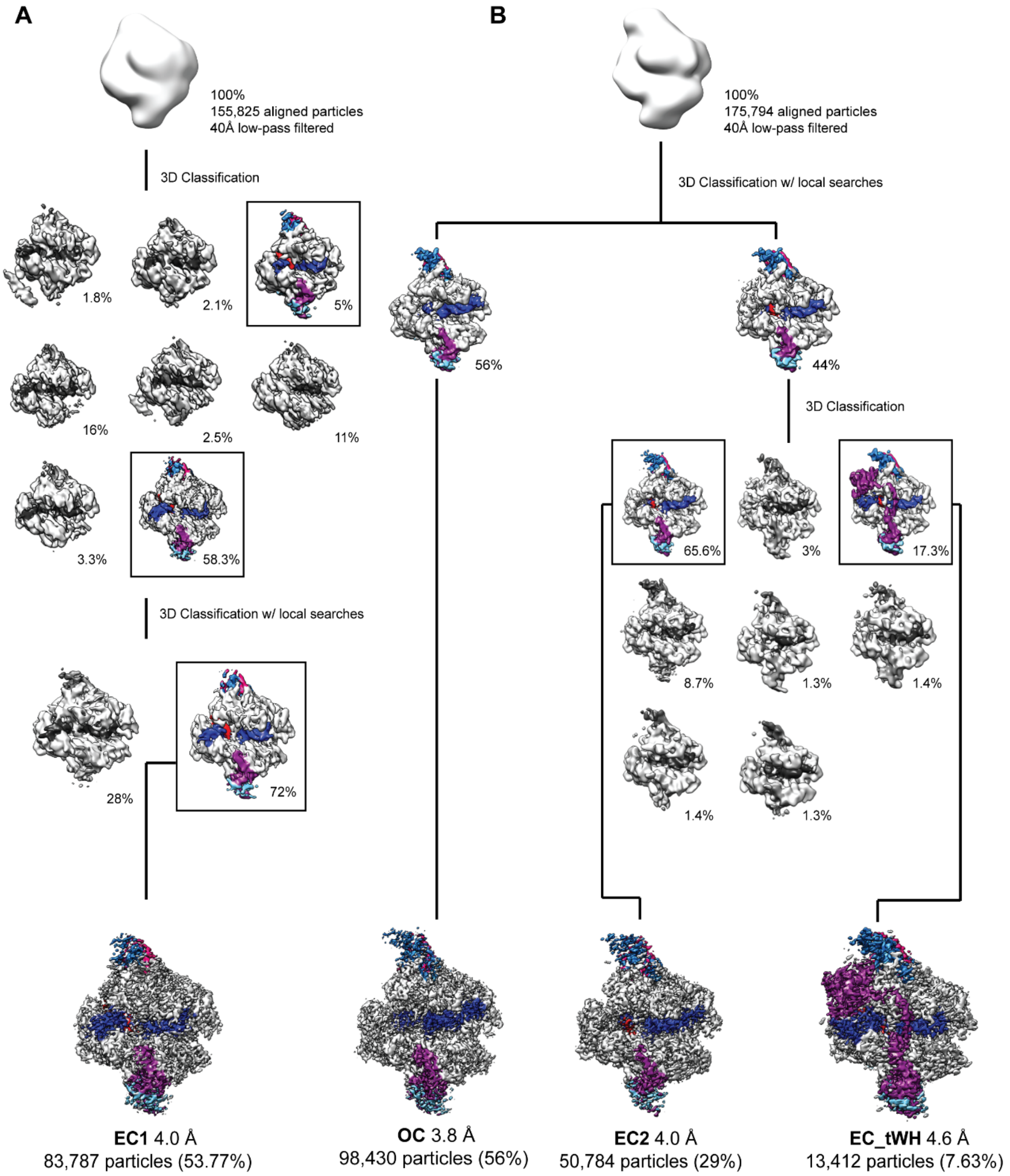

Figure S2. Related to Figure 1. Cryo-EM processing pipeline. A, Processing pipeline for the EC1 dataset. **B**, Processing pipeline for the OC, EC2 and EC_tWH dataset. Density has been colored according to the stalk subunits (A43, marine blue /A14, dark pink), heterodimer subunits (A49, purple /A34.5, lanthanum) and nucleic acids (DNA, blue; RNA, red). The percentage of particles included in each class are shown. Boxed, colored classes indicate classes which were selected for further processing. Discarded classes are shown in grey coloring. The final number of particles and the percentage from the initial aligned particles (100%, shown as the 40Å reference) in each reconstruction are shown.

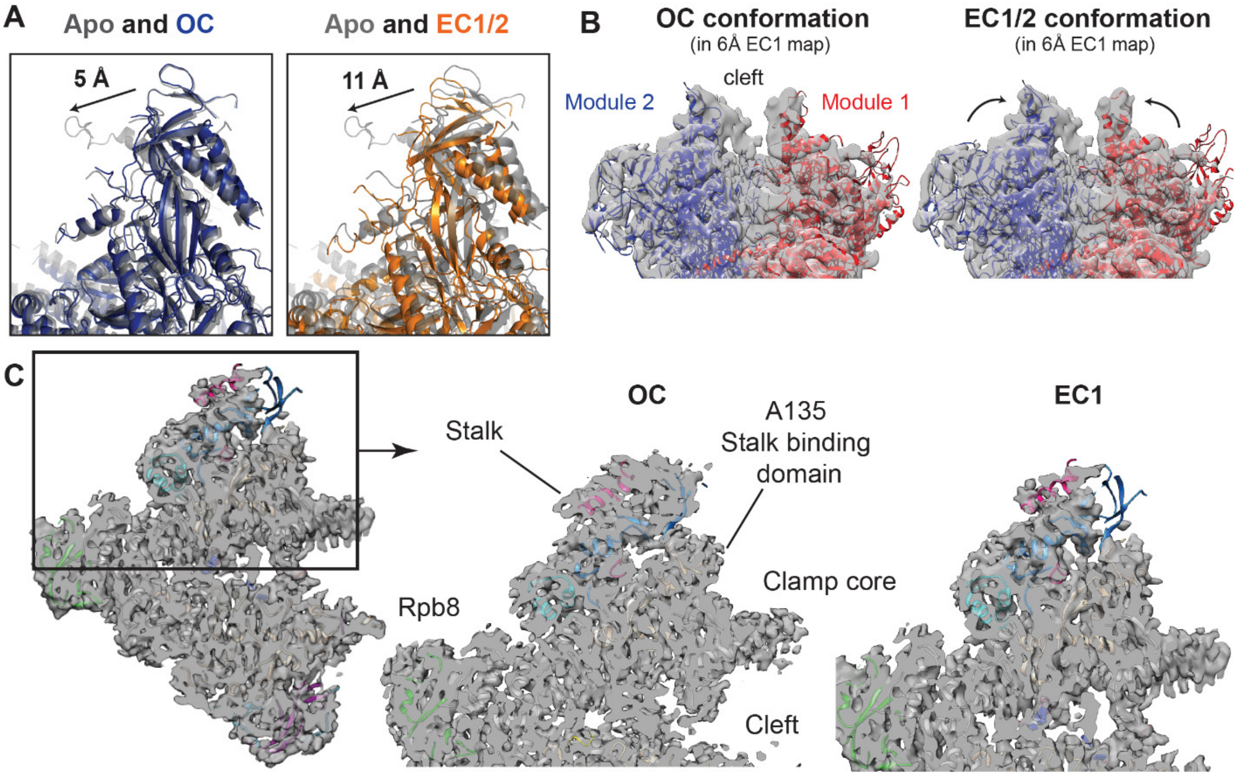

Figure S3. Related to Figure 1. Flexibility of the A43-A14 stalk. **A,** Movement of the stalk is coupled to closing of the cleft. The maximal distance moved relative to apo Pol I is indicated for the OC (left) and EC1/2 (right). **B**, Rigid-body fitting of the OC conformation into the EC1 map, filtered to 6 Å. Module 1 is shown in red and module 2 in blue. Each module fits into the density when moved independently (right) from the OC conformation (left). **C**, A slice of the EM density for the EC1 at a high threshold with the model is shown (left). At this threshold the anchored part of the stalk and the A135 stalk binding domain are seen in the density, but the distal part of the stalk is only visible in the OC. The close-up view of the boxed region is shown for the OC and the EC1 (right).

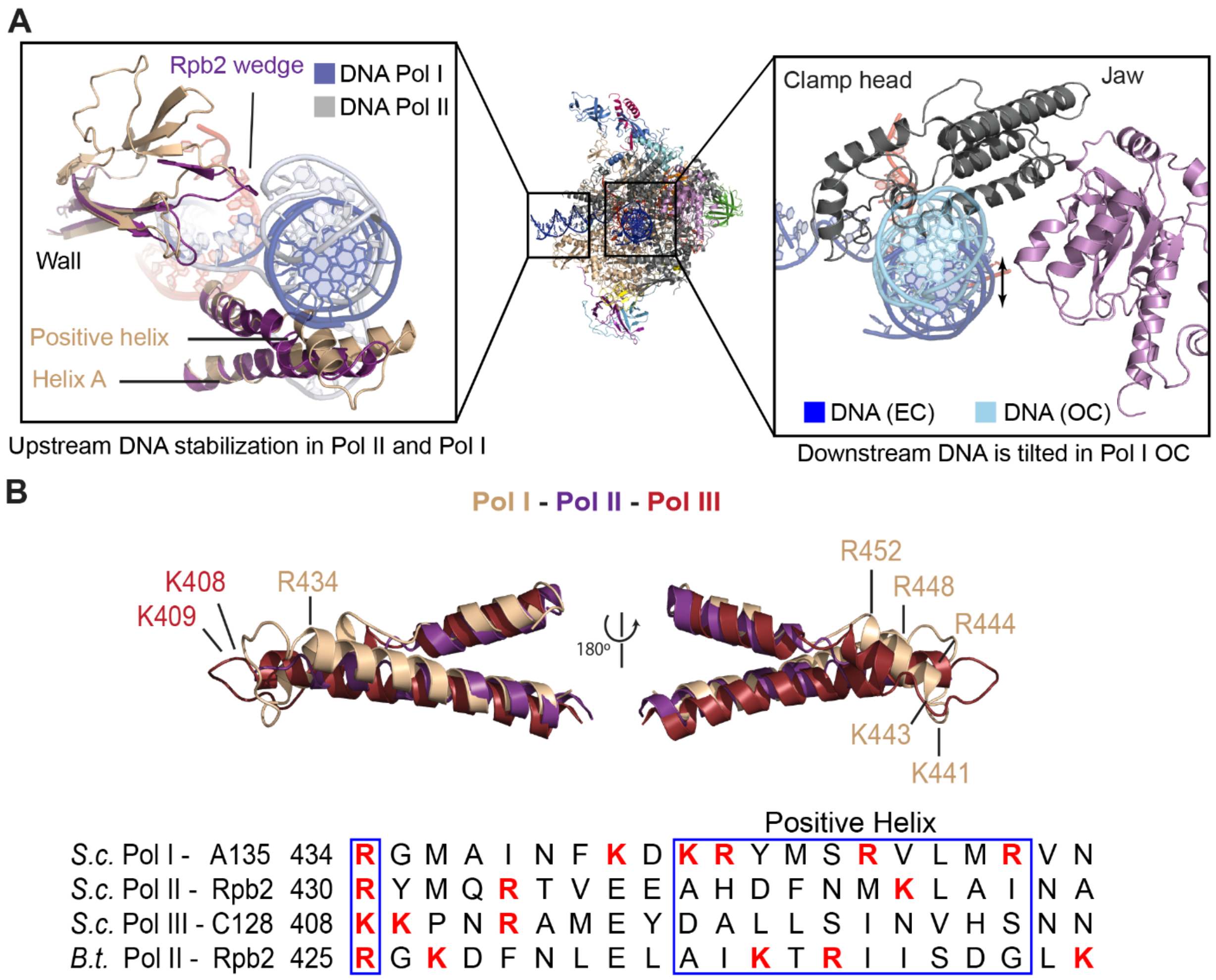

Figure S4. Related to Figure 2. Upstream and downstream DNA contacts in Pol I. A, **Left panel,** compared to Pol II (PDB: 5C4J), the upstream DNA is closer to the protrusion domain than to the wedge. Pol II is indicated in purple, and Pol I A135 subunit is in wheat. Pol I DNA is blue and Pol II DNA is in grey. The protrusion positive helix and helix A are indicated. **Right panel**, The downstream DNA in the OC is tilted towards the A190 clamp head domain and Rpb5 jaw interface, while the downstream DNA in the EC follows a straight path. **B,** Part of the protrusion domain of Pol I, II (PDB: 1Y1W) and III (PDB: 5FJ8) are superimposed. Positive residues in this region are highlighted and the corresponding structure-based sequence alignment is shown below. For comparison, the *Bos Taurus* Rpb2 sequence is aligned, showing that it has more positive residues than *Saccharomyces cerevisiae* Rpb2 and C128 in this region.

Supplemental Experimental Procedures

Protein purification

Pol I was purified from *Saccharomyces cerevisiae* strain SC1613 (also calledYPR110c, provided by Cellzome AG), with a C-terminal TAP-tag fused to AC40. Yeast cells were grown overnight in YPDA medium at 30 ºC and 180 r.p.m. under controlled conditions and collected at an OD_{600} of 5–6. All purification steps were performed at 4 ºC. The cell paste was re-suspended in a buffer containing 250 mM Tris-HCl, pH 7.5, 40% glycerol, 250 mM ammonium sulfate, 1 mM EDTA, 10 mM $MgCl_2$, 10 mM $ZnCl_2$,12 mM β-mercaptoethanol with protease inhibitors (Complete EDTA-free, Roche) and lysed with glass beads in a BeadBeater (BioSpec). The lysate was cleared by centrifugation at 14,000 r.p.m. for 1 h at 4 ºC and loaded on heparin-sepharose resin (GE Healthcare). The complex was eluted from the resin using high-salt buffer with 1 M ammonium sulfate and incubated with IgG Sepharose (GE-Healthcare) for 5 h. After washing the unbound proteins, IgG beads were incubated overnight at 4 ºC with tobacco etch virus (TEV) protease. TAP-tag cleaved Pol I was eluted and further purified by ionic exchange on a Mono-Q column (GE- Healthcare). Pure Pol I enzyme was concentrated and buffer exchanged to 7 mg/ml in 150 mM ammonium sulfate, 15 mM HEPES-NaOH pH 7.5 and 10 mM DTT. Aliquots were stored after flash-freezing in liquid nitrogen or used immediately for complex preparation.

Image processing

All processing steps were performed in RELION-1.4 (Scheres, 2012) unless noted otherwise. The contrast transfer function determination was performed using CTFFIND4 (Rohou and Grigorieff, 2015) and the Thon rings were visually inspected to select good micrographs. B-factor sharpening was performed through implementation in RELION-1.4 as described (Rosenthal and Henderson, 2003) prior to visualization. The resolution is reported according to the FSC 0.143 criterion after particle polishing in RELION. Local resolution of the maps was calculated using Blocres (Cardone et al., 2013).

EC1

Raw movie frames were gain corrected, Fourier cropped, and then aligned using UNBLUR (Grant and Grigorieff, 2015). Initially, approximately 10,000 particles were manually picked using semi-automated particle picking in EMAN2 and extracted with a 200 pixel box size (Tang et al., 2007). Particles were subjected to an initial 2D classification and good classes were selected as templates for the auto-picking procedure in RELION. Autopicked particles (342,189) were subjected to two rounds of 2D classification. Pooled particles (155,825) were aligned using an auto-refine run against the crystal structure of apo Pol I (PDB: 4C3I) low pass filtered to 60 Å and then subjected to 3D classification, using the aligned reconstruction as a reference. Two abundant classes that showed good alignment (98,842 particles, 63.3%) were selected and subjected to 3D classification with restrained angular searches to further sub-classify conformational differences between the retained particles, leading to a class (83,787 particles) which was refined to 4.0 Å. Further classification resulted in maps with the same conformation but lower resolvability. We observe high flexibility for previously known mobile modules. In particular, the A190 Jaw domain, the dimerization interface of the A49-A34.5 heterodimer and the N-terminal region of A12.2 are highly flexible but present in all the reconstruction at lower than the nominal resolution. However, the OB domain of the stalk subunit is completely flexible in the EC1 and could not be resolved by focusing the classification. We attribute this flexibility to movement of the modules during transcription elongation.

OC, EC2, EC_tWH

The movie frames were processed on-the-fly during data acquisition with SerialEM to motion-correct and sum the frames (Li et al., 2015). Approximately 30,000 particles were picked semi-automatically in EMAN2 and extracted with a 176 pixel box size (Tang et al., 2007). Particles were 2D-classified and good classes were used as templates for autopicking. 867,673 autopicked particles were extracted and sorted with 2D classification. Only a minor fraction showed density for CF and Rrn3, while the majority of particles represented Pol I bound to the transcription scaffold. All the particles selected after 2D classification (508,049 particles) were first refined using an auto-refine run against the Apo Pol I crystal structure (PDB: 4C3I) low pass filtered to 40 Å. Subsequently, aligned particles were subjected to 3D classification resulting in one major class (175,794 particles). A next round of 3D classification with restrained angular searches produced two classes: one complex with density for the RNA and a weak extra density (class 1, 44%), and one class without apparent RNA density and with the C-terminal region of

A12.2 (class 2, 56%). The latter was refined to 3.8 Å (OC, 98,430 particles). Further classification of class 2 did not improve the transcription bubble density or resolvability, and no different conformations were observed. Class 1 was further sub-classified and two major classes were refined to 4.0 Å (EC2, 50,784 particles) and 4.6 Å (EC_tWH, 13,412 particles). Additional classification of these classes reduced map quality, probably because of a reduced particle number.

Model building and refinement

For model building of the EC1, EC2 and the OC, the apo Pol I structure (PDB: 4C3I) was used as a starting model. Maps were filtered to different resolution ranges and sharpened with varying B-factors for better interpretation of the density. Subdomains were rigid body fitted in UCSF Chimera (Pettersen et al., 2004) to a 6 Å low pass filtered map. Fitted models were inspected and corrected manually in COOT (Emsley and Cowtan, 2004). For flexible areas in the EC1 (A49-A34.5, A12.2 N-ter, A190 Jaw, distal end of A43-A14), the backbone was rigid body fitted based on the OC or EC2 map which showed better density in these areas and not further built. For the EC_tWH, the crystal structure of the A49 tWH (PDB: 3NFI) and the EC2 model were rigid body fitted in UCSF Chimera to a 8 Å low-pass filtered density. Only three extra residues were built into the tWH crystal structure (residues 182-184). An initial DNA model for the EC1 was built based on a similar transcription bubble (PDB: 5FLM) and modified accordingly. A similar approach was used for building the DNA in the OC, which was used for the EC2 and EC_tWH. Models were real-space refined against the respective maps using a scripted workflow based on CCP4 and PHENIX/cctbx (Adams et al., 2010) libraries essentially as described previously (Fromm et al., 2015; Hoffmann et al., 2015; Hoffmann et al., 2016). Secondary structure restraints were updated at every refinement cycle using conformation analysis based on virtual dihedrals (Williams et al., 2013). We applied additional resolution-dependent restraints for poor map regions using local resolution estimates as described before (Hoffmann et al., 2015). Zinc binding site geometries were restrained to reference distance and angle distributions according to Harding (Harding, 2001; Harding, 2006). Each round of model optimization was evaluated by computing the real-space cross-correlation (RSCC) between experimental map and a map calculated from the model coordinates using B-factor-weighted structure factors from the model coordinates applying electron atomic form factors (Colliex et al, 2006). Individual isotropic atomic displacement parameters (ADPs) were refined by optimizing the real-space correlation between model and experimental map and fitting to the computed RSCC profile. The EC1, EC2, EC_tWH and OC models were refined against the full map reconstructions. We perturbed the models by random atom displacements of 0.4 Å followed by re-refinement against one of the respective half maps (work map). Overfitting of the models was then assessed by calculating the Fourier shell correlation against the work map (FSCwork) and the independent test map (FSCtest). For the OC model, the catalytic loop of A12.2 (residues 99-108) was included in the refinement and deleted afterwards.

Supplemental References

Adams, P.D., Afonine, P.V., Bunkoczi, G., Chen, V.B., Davis, I.W., Echols, N., Headd, J.J., Hung, L.W., Kapral, G.J., Grosse-Kunstleve, R.W.*, et al.* (2010). PHENIX: a comprehensive Python-based system for macromolecular structure solution. Acta Crystallogr D Biol Crystallogr *66*, 213-221.

Cardone, G., Heymann, J.B., and Steven, A.C. (2013). One number does not fit all: mapping local variations in resolution in cryo-EM reconstructions. J Struct Biol *184*, 226-236.

Colliex, C., Cowley, J.M., Dudarev, S.L., Fink, M., Gjønnes, J., Hilderbrandt, R., Howie, A., Lynch, D.F., Peng, L.M., Ren, G., et al. (2006). Electron diffraction. In International Tables for Crystallography (John Wiley & Sons, Ltd).

Fromm, S.A., Bharat, T.A., Jakobi, A.J., Hagen, W.J., and Sachse, C. (2015). Seeing tobacco mosaic virus through direct electron detectors. J Struct Biol *189*, 87-97.

Grant, T., and Grigorieff, N. (2015). Measuring the optimal exposure for single particle cryo-EM using a 2.6 A reconstruction of rotavirus VP6. Elife *4*, e06980.

Harding, M.M. (2001). Geometry of metal-ligand interactions in proteins. Acta Crystallogr D Biol Crystallogr *57*, 401-411.

Harding, M.M. (2006). Small revisions to predicted distances around metal sites in proteins. Acta Crystallogr D Biol Crystallogr *62*, 678-682.

Hoffmann, N.A., Jakobi, A.J., Vorlander, M.K., Sachse, C., and Muller, C.W. (2016). Transcribing RNA polymerase III observed by electron cryomicroscopy. FEBS J *283*, 2811-2819.

Li, X., Zheng, S., Agard, D.A., and Cheng, Y. (2015). Asynchronous data acquisition and on-the-fly analysis of dose fractionated cryoEM images by UCSFImage. J Struct Biol *192*, 174-178.

Rohou, A., and Grigorieff, N. (2015). CTFFIND4: Fast and accurate defocus estimation from electron micrographs. J Struct Biol *192*, 216-221.

Rosenthal, P.B., and Henderson, R. (2003). Optimal determination of particle orientation, absolute hand, and contrast loss in single-particle electron cryomicroscopy. J Mol Biol *333*, 721-745.

Tang, G., Peng, L., Baldwin, P.R., Mann, D.S., Jiang, W., Rees, I., and Ludtke, S.J. (2007). EMAN2: an extensible image processing suite for electron microscopy. J Struct Biol *157*, 38-46.
